# Metformin reduces hepatocarcinogenesis by inducing downregulation of Cyp26a1 and CD8^+^ T cells

**DOI:** 10.1002/ctm2.1465

**Published:** 2023-11-23

**Authors:** Weizhi He, Xicheng Wang, Miaomiao Chen, Chong Li, Wenjian Chen, Lili Pan, Yangyang Cui, Zhao Yu, Guoxiu Wu, Yang Yang, Mingyang Xu, Zhaoxuan Dong, Keming Ma, Jinghan Wang, Zhiying He

**Affiliations:** ^1^ Institute for Regenerative Medicine, Ji'an Hospital, Shanghai East Hospital School of Life Sciences and Technology Tongji University School of Medicine Shanghai China; ^2^ Shanghai Engineering Research Center of Stem Cells Translational Medicine Shanghai China; ^3^ Fudan University Shanghai Cancer Center, International Co‐Laboratory of Medical Epigenetics and Metabolism (Ministry of Science and Technology), Shanghai Medical College of Fudan University, Institutes of Biomedical Sciences Shanghai Key Laboratory of Medical Epigenetics Shanghai China; ^4^ Zhoupu Community Health Service Center of Pudong New Area Shanghai China; ^5^ Postgraduate Training Base of Shanghai East Hospital Jinzhou Medical University Jinzhou Liaoning China; ^6^ Department of Hepatobiliary and Pancreatic Surgery Shanghai East Hospital, Tongji University Shanghai China

**Keywords:** atRA, CD8^+^ T cells, Cyp26a1, hepatocellular carcinoma, metformin

## Abstract

**Background:**

Hepatocellular carcinoma (HCC) is a highly heterogeneous cancer with major challenges in both prevention and therapy. Metformin, adenosine monophosphate‐activated protein kinase (AMPK) activator, has been suggested to reduce the incidence of HCC when used for patients with diabetes in preclinical and clinical studies. However, the possible effects of metformin and their mechanisms of action in non‐diabetic HCC have not been adequately investigated.

**Methods:**

Fah^−/−^ mice were used to construct a liver‐injury‐induced non‐diabetic HCC model for exploring hepatocarcinogenesis and therapeutic potential of metformin. Changes in relevant tumour and biochemical indicators were measured. Bulk and single‐cell RNA‐sequencing analyses were performed to validate the crucial role of proinflammatory/pro‐tumour CD8^+^ T cells. In vitro and in vivo experiments were performed to confirm Cyp26a1‐related antitumour mechanisms of metformin.

**Results:**

RNA‐sequencing analysis showed that chronic liver injury led to significant changes in AMPK‐, glucose‐ and retinol metabolism‐related pathways in Fah^−/−^ mice. Metformin prevented the formation of non‐diabetic HCC in Fah^−/−^ mice with chronic liver injury. *Cyp26a*1 dd*expression* in hepatocytes was significantly suppressed after metformin treatment. Moreover, downregulation of Cyp26a1 occurred in conjunction with increased levels of all‐trans‐retinoic acid (atRA), which is involved in the activation of metformin‐suppressed hepatocarcinogenesis in Fah−/− mice. In contrast, both CD8^+^ T‐cell infiltration and proinflammatory/pro‐tumour cytokines in the liver were significantly upregulated in Fah^−/−^ mice during chronic liver injury, which was notably reversed by either metformin or atRA treatment. Regarding mechanisms, metformin regulated the decrease in Cyp26a1 enzyme expression and increased atRA expression via the AMPK/STAT3/Gadd45β/JNK/c‐Jun pathway.

**Conclusions:**

Metformin inhibits non‐diabetic HCC by upregulating atRA levels and downregulating CD8^+^ T cells. This is the first reporting that the traditional drug metformin regulates the metabolite atRA via the Cyp26a1‐involved pathway. The present study provides a potential application of metformin and atRA in non‐diabetic HCC.

## Graphical abstract

1. Metformin inhibits non‐diabetic HCC induced by chronic liver injury.
2. Metformin decreases Cyp26a1 and thereafter increases the level of atRA.
3. Pro‐tumour CD8^+^ T cells are increased significantly during hepatocarcinogenesis, and both metformin and atRA reverse this effect.
4. AMPK pathway is suppressed during tumour formation and metformin decreases Cyp26a1 expression through modulating AMPK/STAT3/Gadd45β/JNK/c‐Jun pathway.

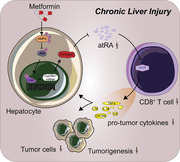

## 1 BACKGROUND

Hepatocellular carcinoma (HCC) is the sixth‐most common neoplasm and the third leading cause of cancer‐related deaths worldwide.^1^ Although great advances have been made in HCC treatment over the past few decades, the rate of HCC mortality remains very high. HCC is a complex malignancy that can be triggered by various factors, including hepatic viral infections, environmental exposure to toxic substances (such as aflatoxin B1), alcohol abuse and non‐alcoholic fatty liver disease (NAFLD).^2^ Moreover, growing evidence supports an association between HCC and metabolic syndrome, diabetes and obesity.^3^ In addition to these acquired forms of HCC, patients with certain genetic defects are also likely to develop HCC.^4^, ^5^, ^6^ The therapeutic approach for HCC is complicated by its heterogeneity, especially its diverse causes and modes of progression.

Metformin (Met), a synthetic guanidine analogue, is the most commonly prescribed drug for treating type 2 diabetes mellitus.^7^ In addition to reducing glucose levels, metformin directly inhibits complex I of the electron transport chain, resulting in decreased complex I activity and oxidative phosphorylation level.^8^, ^9^, ^10^ Consequently, an elevated adenosine monophosphate/adenosine triphosphate (AMP/ATP) ratio activates the AMP‐activated protein kinase (AMPK) signalling pathway, which promotes cell cycle arrest and inhibits tumour cell proliferation.^11^ Recent studies have demonstrated that clinical doses of metformin‐bound PEN2 form a complex with ATP6AP1, which leads to the inhibition of v‐ATPase and activation of AMPK without affecting cellular AMP levels.^12^ Preclinical and retrospective population‐based studies have demonstrated the antitumour activity of metformin alone or in combination with conventional anticancer drugs.^13^, ^14^, ^15^, ^16^ Preclinical and clinical studies have also suggested that in patients with diabetes, prolonged use of metformin could be associated with a reduction in the incidence of HCC.^17^, ^18^ Several research groups have demonstrated that metformin inhibits non‐diabetes‐induced HCC in mouse models.^19^, ^20^ Williams et al. showed that metformin prevents HCC development by alleviating p21 overexpression and ameliorating the pro‐tumourigenic microenvironment.^19^ Shankaraiah et al. demonstrated that metformin prevents hepatocarcinogenesis by attenuating fibrosis in a transgenic mouse model of HCC.^20^ However, despite decades of research, the mechanism underlying the HCC‐inhibiting effects of metformin remains a topic of debate. This lack of clarity and the heterogeneity of HCC encouraged us to determine the effects and mechanism of metformin in chronic liver injury (CLI) models of HCC.

Hereditary tyrosinemia type 1 (HT1), which is caused by a deficiency in fumarylacetoacetate hydrolase (FAH),^21^ is a severe inherited disorder of tyrosine degradation. FAH deficiency leads to accumulation of toxic metabolites, mainly in the liver.^22^ Untreated HT1 patients usually die before 2 years of age. HT1 patients experience multiple chronic complications, including cirrhosis and a high risk of HCC.^22^ Nitisinone (NTBC; a drug that blocks the pathway upstream of FAH) greatly delays the morbidity of HT1 patients, but patients receiving NTBC may still develop HCC.^23^ However, the molecular basis of HT1 pathogenesis remains unclear. The murine model of Fah deficiency (Fah*^−/−^*) is a suitable animal model that features all the phenotypic and biochemical expressions of HT1 patients.^24^ Moreover, tumours generated under particular conditions of induced liver injury in mice with an FAH‐deficient genetic background closely resemble human HCC under alcohol‐induced and c‐Myc‐altered conditions.^25^ Therefore, FAH‐deficient, chronically injured mice may serve as a good model for studying human HCC.

In this study, we demonstrated that several unique signalling pathways were significantly altered in an HCC model induced by CLI. These pathways are characterised by the regulation of glucose and retinol metabolism. Metformin increased AMPK activity and subsequently reduced the incidence of HCC. Our data also showed that metformin suppressed the Cyp26a1 enzyme, leading to an increase in all‐trans‐retinoic acid (atRA) levels, and reducing CD8^+^ T‐cell infiltration and proinflammatory/pro‐tumour cytokines secreted by CD8^+^ T cells. Moreover, metformin suppressed Cyp26a1 expression through the AMPK/STAT3/Gadd45β/JNK/c‐Jun pathway. Our results are the first to reveal a regulatory link between metformin and atRA, which may explain the similarity of their antitumour activities by inhibiting pro‐tumour CD8^+^ T cells.

## 2 METHODS

### 2.1 Patient specimens

Patient specimens were obtained from the sample information service platform of Shanghai East Hospital under protocols approved by Shanghai East Hospital. The final diagnoses of the specimens were confirmed by experienced pathologists from Shanghai East Hospital. The study protocol conformed to the ethical guidelines approved by the Shanghai East Hospital Ethics Committee, and written informed consent was obtained from each patient.

### 2.2 Analysis of public clinical datasets

The gene expression data of 7858 samples from 30 normal tissues (including liver tissues) were downloaded from GTEx (https://gtexportal.org/home/), and the gene expression data of 710 samples from 17 para‐tumour tissues and 7801 samples from 34 cancer types (including HCC) were downloaded from The Cancer Genome Atlas (TCGA; https://portal.gdc.cancer.gov/). Correlation analyses between genes and signatures were performed using the GEPIA web server (http://gepia.cancer‐pku.cn).^26^

We also obtained mRNA expression data from CLI cases using GSE89632 and GSE148355. To determine whether the AMPK signalling pathway is inhibited in human CLI as in the mouse model, we downloaded the RNA‐sequencing (RNA‐seq) dataset GSE148355^27^ and the microarray dataset GSE89632^28^ from the GEO public database for verification. The GSE148355 dataset was composed of HCC samples, ‘premalignant’ samples with different degrees of fibrosis, and non‐tumour samples from patients who had undergone surgical resection. We mainly used the normalised data (FPKM, Fragments Per Kilobase of exon model per Million mapped fragments) of 62 premalignant and non‐tumour samples for analysis, including 15 non‐tumour samples (normal), 10 low‐fibrosis samples (fibrosis low), 10 high‐fibrosis samples (fibrosis high), 10 cirrhosis samples (cirrhosis), 10 samples of nodules with low‐degree dysplasia (dysplastic nodule low) and seven samples of nodules with high‐degree dysplasia (dysplastic nodule high). We also analysed microarray data from 63 NAFLD and healthy samples from the GSE89632 dataset, including 20 simple steatosis (SS), 19 non‐alcoholic steatosis hepatitis (NASH) and 24 healthy control (HC) samples.

In this study, we used the key genes of the AMPK pathway to form a self‐defined functional gene set, which was composed of *PFKFB3*, *CPT1A*, *SIRT1*, *PPARGC1A*, *SLC2A4*, *PNPLA2*, *CRY1* and *FOXO1*. We also collected and used the key genes of the atRA pathway to form a self‐defined functional gene set, which was composed of *HOXA1*, *CDX1*, *ARG1*, *DHRS3*, *LHX1*, *PTPRZ*, *GCNF1*, *MRG1*, *NRIP1*, *ALDH1A1*, *ALDH1A2*, *ALDH1A3*, *RXRA*, *RXRB*, *RXRG*, *RARA*, *RARB*, *RARG*, *RDH16*, *RDH10*, *DHRS9* and *CES1*. To infer whether the AMPK pathway is inhibited during CLI, a single‐sample gene set enrichment analysis (ssGSEA) algorithm was used to calculate the enrichment score of this gene set in normal samples and samples with different degrees of CLI.^29^ The expression of these eight genes was statistically analysed in both healthy and CLI samples.

### 2.3 Mouse model and treatments

As described in previous studies,^30^, ^31^ 129S4 Fah^−/−^ mice undergo liver failure and death. All mice were bred and maintained under specific pathogen‐free conditions at the Shanghai East Hospital. The mice were housed in a light:dark cycle of 12 h, ambient temperature of 24°C, and humidity of 55%. Then, 7.5 mg/L of NTBC was supplemented to the drinking water for pre‐experiment, which was used to bred Fah^−/−^ mice to block the occurrence of liver injury, and we reduced the dosage of NTBC to 2.5% (.1875 mg/L) for the induction of CLI. Female mice were randomly allocated to either the metformin/atRA‐treated or untreated groups. Mice in the metformin experiments received metformin (B1970, APExBIO) via intraperitoneal injection at a dose of approximately 250 mg/kg/day from 0 to 12 weeks of CLI (under 2.5% NTBC), while those in the atRA experiments received 200 μg of atRA (Sigma–Aldrich) dissolved in dimethyl sulphoxide (DMSO) or DMSO alone by intraperitoneal injection every other day from 0 to 12 weeks of CLI (under 2.5% NTBC). Fah^−/−^ mice with chronic injury for 12 weeks were treated with oral 2.5 mg/kg/day talarozole (Talarozole HY‐14531 from MedChemExpress in 10% DMSO, 40% Polyethylene glycol 300, 5% Tween‐80, 45% saline) or solvent alone for another 4 weeks. We used ‘resource equation of sample size calculation’ to calculate the minimal number of animals that were used for statistical significance.^32^ In our study, the number of animals we used (*n* = 12) was more than the minimal number of animals (*n* = 6) used for statistical significance. All experimental procedures involving mice were approved by the Institutional Animal Care and Use Committee of the Shanghai East Hospital.

### 2.4 Quantitative real‐time polymerase chain reaction

Quantitative real‐time polymerase chain reaction (qRT‐PCR) was performed as described previously.^30^ Briefly, total RNA was isolated from the liver of mice using RNAiso Plus (TaKaRa). RNA was reverse‐transcribed using the PrimeScript RT Reagent Kit with gDNA Eraser (TaKaRa Bio). Transcript expression was determined by qRT‐PCR using SYBR Premix Ex Taq II (TaKaRa) and a QuantStudio real‐time PCR instrument (Applied Biosystems). The primer sequences used were as follows: Cyp26a1: 5′‐AAGCTCTGGGACCTGTACTGT‐3′ and 5′‐CTCCGCTGAAGCACCATCT‐3′, *Gapdh*: 5′‐AGGTCGGTGTGAACGGATTTG‐3′ and 5′‐TGTAGACCATGTAGTTGAGGTCA‐3′, *Il6*: 5′‐GTCCTTCCTACCCCAATTTCC‐3′ and 5′‐TAACGCACTAGGTTTGCCGA‐3′, *Il1β*: 5′‐GCAACTGTTCCTGAACTCAACT‐3′ and 5′‐ATCTTTTGGGGTCCGTCAACT‐3′, *Tnf*: 5′‐CCCTCACACTCAGATCATCTTCT‐3′ and 5′‐GCTACGACGTGGGCTACAG‐3′, *Ltβ*: 5′‐TGGCAGGAGCTACTTCCCT‐3′ and 5′‐TCCAGTCTTTTCTGAGCCTGT‐3′, *Gadd45β*: 5′‐CGGAGACATTGGGCACAAC‐3′ and 5′‐CCTTGGCTTTTCCAGGAATCT‐3′. In designing the primers, most primers used in our study are in the spam exon junction, which is for reducing the genomic DNA contamination. qRT‐PCR was performed using the comparative CT method. The data were presented either as fold changes of the treated group in comparison with the control group or as 2^−ΔΔCT^ mRNA transcript abundance.

### 2.5 RNA‐seq and transcriptomic analyses of mouse liver tissues

Total RNA (two mouse liver tissue samples from both C0 and C4 and three liver tissue samples from both C12 and Met12) was extracted using the mirVana miRNA Isolation Kit (Ambion) in accordance with the manufacturer's protocol. RNA integrity was evaluated using the Agilent 2100 Bioanalyser (Agilent Technologies). Samples with an RNA integrity number ≥7 were subjected to subsequent analyses. Libraries were constructed using the TruSeq Stranded mRNA LT Sample Prep Kit (Illumina) according to the manufacturer's instructions. These libraries were sequenced on an Illumina sequencing platform (HiSeqTM 2500 or Illumina HiSeq X Ten), and 125‐bp/150‐bp paired‐end reads were generated.

Transcriptome sequencing and analysis were performed by OE Biotech Co., Ltd. Raw data (gene sequencing reads) were processed using Trimmomatic software.^33^ Reads containing poly‐N and low‐quality reads were removed to obtain clean reads. The clean reads were mapped to the reference genome GRCm38.p6 (http://ncbi.nlm.nih.gov/genomes/all/GCF/000/001/635/GCF_000001635.26_GRCm38.p6/GCF_000001635.26_GRCm38.p6_genomic.fna.gz) using hisat2.^34^ The FPKM value of each gene was calculated using Cufflinks, and the read counts of each gene were obtained by evaluating the htseq count.^35^ Differentially expressed genes (DEGs) were identified using the DESeq (2012) R package functions to estimate the size factors and nbinomTest. A *p*‐value <.05 and fold change >2 or <.5 were set as the thresholds for significantly different expression. Hierarchical cluster analysis of DEGs was performed to explore gene expression patterns. Gene Ontology (GO) enrichment and Kyoto Encyclopedia of Genes and Genomes (KEGG) pathway enrichment analysis of DEGs were performed using the DAVID web server (https://david.ncifcrf.gov/).^36^, ^37^

To understand the mechanisms underlying the HCC‐preventing effects of metformin, we also downloaded the GSE110524 data from the study by Williams et al., in which metformin prevented HCC development in Ncoa5^+/−^ mice CLI.^19^ The batch effect was removed using the ‘combat’ function in sva package,^38^ and comprehensive combination analyses were conducted, including principal component analysis (PCA), clustering analysis, differential analysis (limma package, version 3.50.1^39^) as well as a correlation analysis in R. Since the two combination analyses of metformin treatment in different mouse models may provide clues in realising the potential mechanism for both treating and preventing hepatocarcinogenesis, weighted gene co‐expression network analysis (WGCNA) with the default parameters was used to identify key gene modules for metformin.^40^

### 2.6 Protein isolation and western blot analyses

Livers were harvested from normal Fah^−/−^ mice (100% NTBC), Fah^−/−^ mice with CLI (2.5% NTBC) or mice at different stages of HCC development. Lysates were prepared from liver tissues using RIPA lysis buffer. The following primary antibodies were used for immunoblotting: anti‐GAPDH (Proteintech, HRP‐60004), anti‐AMPK (CST, 2532), anti‐p‐AMPK (CST, 2535), anti‐CYP26A1 (Santa Cruz, sc‐53618), anti‐p‐JNK (CST, 9255), anti‐JNK (CST, 9252), anti‐p‐c‐Jun (Abcam, ab40766) and anti‐c‐Jun (Abcam, ab32385). Primary and secondary horseradish peroxidase (HRP)‐labelled antibodies were used at dilutions of 1:500 and 1:2000, respectively. Detection was performed using the SuperSignal West Femto Maximum Sensitivity Substrate (Thermo Scientific).

### 2.7 Metformin treatment of primary hepatocytes

Livers of Fah^−/−^ mice with CLI were perfused as previously described.^41^ Primary hepatocytes (5 × 10^5^) were seeded in a six‐well plate (Matrigel coated) with 2 mL of advanced DMEM/F‐12 (Thermo Fisher, 12634010) supplemented with 10% foetal calf serum. The old medium was replaced with fresh medium after 24 h. Cells were treated with different concentrations of metformin or 5‐aminoimidazole‐4‐carboxamide1‐β‐D‐ribofuranoside (AICAR) for 6 h. Hepatocytes were pretreated with the inhibitor (Compound C, SP600125 or stattic) for 1 h, followed by the addition of metformin.

### 2.8 Luciferase assay

Chronically injured livers from Fah^−/−^ mice were perfused. Approximately 2 × 10^4^ primary hepatocytes were seeded in a 24‐well plate with 1 mL of advanced DMEM/F‐12 supplemented with 10% foetal calf serum. Two hours later, 1 mL of advanced DMEM/F‐12 without foetal calf serum was used to replace the old medium. On the second day, the cells were transfected with a 6:1 ratio of firefly luciferase reporter plasmid driven by a pGL3‐RARE‐responsive promoter (Addgene, plasmid #13458) and a Renilla luciferase reporter plasmid driven by a constitutive CMV promoter (Promega). After 24 h, the culture medium was replaced with fresh advanced DMEM/F‐12 medium, metformin/atRA or vehicle. After 24 h, the activity of both reporters was measured using a Dual‐Luciferase Reporter kit (Promega) and read on a Tecan Infinite 200 PRO Reader. The firefly luciferase‐to‐Renilla luciferase ratio is reported as ‘firefly/Renilla luciferase activity’.

### 2.9 Chromatin immunoprecipitation PCR

Chromatin immunoprecipitation (ChIP) was performed using the SimpleChIP Enzymatic Chromatin IP Kit (Magnetic Beads) (CST# 9003). Briefly, primary mouse hepatocytes treated with or without metformin (4 mM) for 6 h were treated with 1% formaldehyde at room temperature for 10 min, followed by the addition of glycine to quench the unreacted formaldehyde. Cells were lysed, and cross‐linked DNA was enzymatically sheared to approximately 200–800 bp. Protein and DNA complexes were precipitated using specific antibodies against c‐Jun (CST# 9165) and the immunoglobulin G control (CST# 2729). NaCl was added to lysates to reverse DNA–protein crosslinks, which was followed by incubation at 65°C for 4–5 h. ChIP‐enriched chromatin was used for qPCR with SYBR Green Master Mix. Specific primer sequences for the predicted binding sites of mouse Cyp26a1 promoter were as follows: Cyp26a1 chip primer F: 5′‐GAGAAGGGAGTCAGGCATGT‐3′; Cyp26a1 chip primer R: 5′‐AAACGCTTCCTGATCTGGGA‐3′.

### 2.10 Serological analyses

For evaluation of serum indicators, blood samples were collected from the retroorbital sinuses of the test animals. Plasma was prepared using microtainer plasma separator tubes (BD) and stored at −80°C. Serum biochemical indicators were analysed according to a previously established protocol.^30^

### 2.11 Haematoxylin–eosin staining and immunohistochemical analysis

Haematoxylin and eosin (H&E) staining and immunohistochemistry (IHC) were performed as described previously.^30^ For H&E staining, fresh liver tissues were fixed with 4% paraformaldehyde in phosphate‐buffered saline, routinely embedded in paraffin, and sectioned into slices (2 μm). The slices were processed for roasting, dewaxing and rehydration, stained with haematoxylin (Beyotime) for 5−10 min, rinsed with water for 15 min, dehydrated in 95% alcohol (Sinoreagent) for 30 s and stained with eosin (Beyotime) for an appropriate amount of time (.5−2 min). Finally, the slices were rapidly dehydrated and mounted using a neutral resin (MXB).

For IHC staining, the detailed steps were the same as those for H&E staining before rehydration. The slices were then soaked in .01 M citric acid buffer (pH 6.0) or EDTA (Ethylenediaminetetraacetic acid, pH 9.0) and placed in a pressure cooker for 2−4 min at 121°C/100 kPa. The solution was cooled to room temperature. Endogenous peroxidase was blocked with 3% H_2_O_2_ solution, and 1% bovine serum albumin was used to block nonspecific loci for 30 min at room temperature. The slices were incubated with the primary antibodies at 4°C overnight and HRP‐conjugated secondary antibodies at 37°C for 30 min. DAB staining (Vector Laboratories) was applied to the sections. Sections were stained with haematoxylin (Beyotime), rapidly dehydrated and mounted with neutral resin (MXB). Primary antibodies against CD8 (Abcam, ab209775) and CD45 (CST, 70257) were used.

### 2.12 Flow cytometry

Fresh liver tissue samples were minced and digested using the mouse Liver Dissociation Kit (MACS 130‐105‐807) for .5 h at 37°C. Cell suspensions were generated using a 70 μm nylon mesh. After filtration and washing, the cells were suspended in 40% Percoll solution and centrifuged at 600 × *g* for 20 min. Cells were prepared as single‐cell suspensions for FACS (Fluorescence activated cell sorting) staining. The following antibodies were used: APC‐CY7‐LIVE/DEAD (BD, 565388), FITC‐CD45 (BD, 553099), V450‐CD3 (BD, 560801), AF700‐CD4 (BD, 557956), V500‐CD8 (BD, 560776), BV605‐CD49B (BD, 740363), PE‐F4/80 (BD, 565410) and BB700‐CD11B (BD, 566416). The stained cells were analysed using Cytoflex (BECHMAN). Flow cytometry data were analysed using FlowJo software (Tree Star Inc.).

### 2.13 Blood glucose measurement

Blood was sampled from mice by nicking the tail vein, and blood glucose levels were measured using ACCU‐CHEK Active test strips read using an ACCU‐CHEK Active meter (Roche Diagnostics) in accordance with the manufacturer's instructions.

### 2.14 Single‐cell RNA‐seq analysis

The single‐cell RNA‐seq (scRNA‐seq) data of Fah*^−/−^* mice at 0, 12 and 18 weeks after NTBC withdrawal were downloaded from the GEO database with the GEO accession number GSE130880.^42^ In addition, scRNA‐seq data of NASH mice capturing the progression of NAFLD pathogenesis with a high‐fat high‐fructose diet for 15 weeks (liver injury) and 30 and 34 weeks (severe fibrosis and inflammation)^43^ were also obtained. Dimension‐reduction analyses of T‐distributed stochastic neighbour embedding and uniform manifold approximation and projection were performed using the Seurat package (R‐version 4.0.5). Correlation analyses between the signatures were performed to understand the relationship between functional signatures and genes. In addition, we used the ssGSEA algorithm^29^ to calculate the scores of the pro‐tumour CD8^+^ T‐cell signature in CLI Fah*^−/−^* mice at 0, 12 and 18 weeks. The single‐cell regulatory network inference and clustering (SCENIC) package was used to confirm potential regulation between the AMPK pathway and c‐Jun in hepatocytes.^44^

### 2.15 Statistical analyses

All results are presented as the mean ± standard deviation as indicated. Differences between groups were analysed using one‐way or two‐way analysis of variance, Student's *t*‐test or two‐tailed Mann–Whitney *U*‐test. Overall survival and disease‐free survival were analysed using the log‐rank test with GraphPad Prism 7. The following symbols were used to indicate statistical significance: ^*^ *p* < .05, ^**^ *p* < .01, ^***^ *p* < .001, NS: not significant.

## 3 RESULTS

### 3.1 Characteristics of tumour formation in the livers of Fah^−/−^ mice with CLI

Using the Fah*^−/−^* mouse model as a surrogate for chronic liver disease‐induced HCC, we first described the liver phenotype of Fah*^−/−^* mice under different conditions using the findings obtained from our study and other published studies.^30^, ^45^ As described in Figure S1, at 100% concentration of NTBC (7.5 mg/L), the liver of Fah*^−/−^* mice was preserved normally; Fah*^−/−^* mice developed acute liver injury and died at 3−6 weeks without any NTBC; and at 2.5% concentration of NTBC (.2 mg/L), Fah*^−/−^* mice survived but suffered CLI and showed HCC after 12 weeks.

**FIGURE 1:**
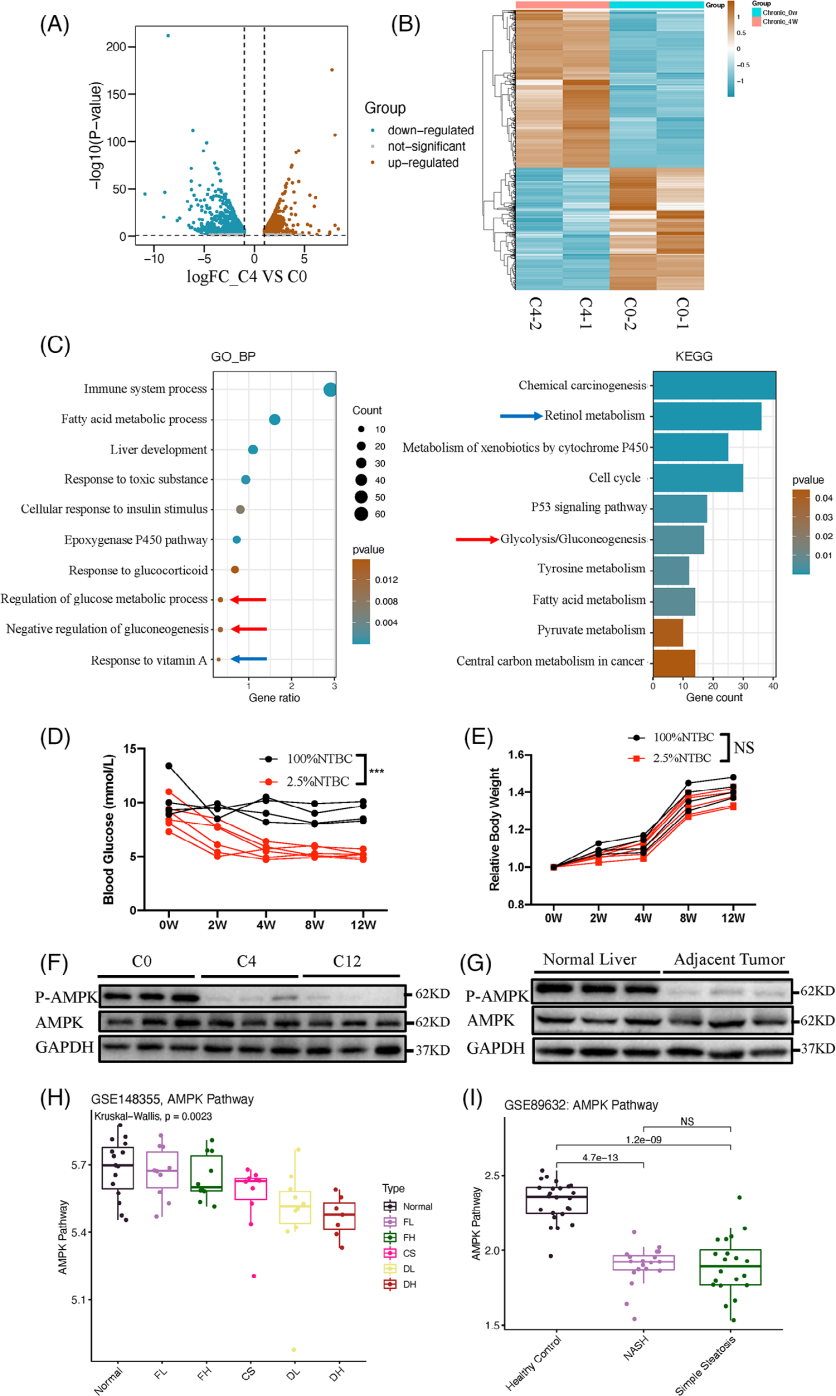
Characteristics of Fah^−/−^ mice with chronic liver injury. (A) RNA‐sequencing showing volcano plot of differential expression analysis results from 4 weeks of chronic liver injury (C4) versus normal Fah^−/−^ mouse liver (C0). (B) Heatmap illustrating differential gene expression of liver tissues from 4 weeks chronic liver injury (C4, labelled as FT4 here) versus normal Fah^−/−^ mouse (C0, labelled as FT0 here). (C) Gene Ontology (GO) classification and Kyoto Encyclopedia of Genes and Genomes (KEGG) pathway enrichment analyses of the differentially expressed genes from 4 weeks of chronic liver injury (C4) versus normal Fah^−/−^ mouse liver (C0). (D) Body weight (BW) in Fah^−/−^ mice with chronic liver injury (2, 4, 8 and 12 weeks) and normal Fah^−/−^ mouse (0 week). (E) Blood glucose levels in Fah^−/−^ mice with chronic liver injury (2, 4, 8 and 12 weeks) and normal Fah^−/−^ mouse (0 week). (F) Western blot analysis of adenosine monophosphate‐activated protein kinase (AMPK) activity from 4 weeks (C4), 12 weeks (C12) chronic liver injury and normal Fah^−/−^ mouse liver (C0). (G) Western blot analysis of P‐AMPK level in healthy human liver tissues and adjacent noncancerous liver tissues of hepatocellular carcinoma (HCC) patients. (H) Enrichment score of AMPK pathway in normal and premalignant tissues. The enrichment score of AMPK pathway was calculated by single‐sample gene set enrichment analysis (ssGSEA) algorithm. (I) Enrichment score of AMPK pathway in normal tissues and non‐alcoholic fatty liver disease (NAFLD) tissues. Normal: non‐tumour normal control, FL: low fibrosis, FH: high fibrosis, CS: cirrhosis, DL: dysplastic nodule low, DH: dysplastic nodule high and NASH: non‐alcoholic steatohepatitis. All data are represented as the mean ± standard deviation (SD). Statistical significance was determined by unpaired ordinary analysis of variance (ANOVA). ^*^ *p* < .05; ^**^ *p* < .01; ^***^ *p* < .001.

To gain further insight into the molecular mechanisms that contribute to HCC development in CLI, we first aimed to characterise the dynamic transcriptomic changes in chronically injured livers in comparison with normal livers. After high‐throughput RNA‐seq, the DEGs between the normal livers of Fah*^−/−^* mice (100% NTBC, named C0) and chronically injured livers of Fah*^−/−^* mice (2.5% NTBC for 4 weeks, named C4) were identified (Figure 1A,B and Table S1). Two thousand genes with significantly altered expression were identified (log2‐fold change > 1, *p* < .05). Genes with expression levels altered by more than twofold were further analysed using gene enrichment analyses, including GO and KEGG analyses. Several pathways had been enriched in Fah*^−/−^* mice with CLI (Fah*F^−/−^* mice) (Figure 1C and Table S2). HCC is a classic instance of inflammation‐related cancer, and chemically or genetically induced HCC is highly dependent on inflammatory signalling.^3^ We found that many of these DEGs were involved in inflammatory processes, and were also implicated in the progression from a normal status to CLI and finally to HCC (Figure 1C). Consistently, several KEGG pathway terms, especially the p53 signalling pathway, were altered in Fah*^−/−^* mouse livers (Figure 1C). The process of retinol metabolism was also significantly altered in both CLI and normal Fah*^−/−^* mice (Figure 1C, blue arrow). Notably, GO and KEGG analyses also showed that the glucose metabolic process was enriched in the CLI Fah*^−/−^* mice (Figure 1C, red arrow). Body weight and blood glucose level in the CLI Fah*^−/−^* mice were measured. In comparison with normal Fah*^−/−^* mice, CLI Fah*^−/−^* mice showed similar body weight and significantly lower blood glucose level, indicating that HCC formation in CLI Fah*^−/−^* mice represents non‐diabetes‐induced HCC (Figure 1D,E). The effects of metformin depend on the activation of AMPK^12^; therefore, we aimed to determine whether AMPK activity was altered in our CLI model of Fah*^−/−^* mice. We evaluated AMPK activity in Fah*^−/−^* mouse livers of C0 (uninjured control, 0 weeks), C4 (CLI, 4 weeks) and C12 (CLI, 12 weeks) mice, and found that AMPK activity in the liver decreased significantly when Fah*^−/−^* mice showed CLI (Figure 1F).

In parallel, we tested human patients with normal or cirrhotic liver tissues and found that AMPK activity was also repressed in chronically injured human livers (Figure 1G). To understand whether the inhibition of AMPK signalling showed a similar trend in human CLI and the mouse model, we obtained two human CLI‐related datasets from the GEO database, and the ssGSEA algorithm was applied to evaluate whether the AMPK pathway was hampered under CLI. With the aggravation of liver fibrosis, the enrichment score of the AMPK pathway gradually declined, and the AMPK pathway tended to be suppressed in all chronically injured liver tissues in comparison with normal tissues (Figure 1H). Similarly, the enrichment scores of the AMPK pathway in the NASH and SS groups were significantly lower than those in the HC group (Figure 1I). In addition, we observed that the expression levels of *FOXO1*, *CRY1* and other AMPK pathway core genes were significantly lower in chronically injured livers than in HCs (Figure S2). In addition, preclinical and clinical studies have suggested that AMPK activity in the human liver decreases significantly with CLI, and lower AMPK activity indicates poorer survival.^46^, ^47^, ^48^, ^49^ These data indicate that CLI Fah*^−/−^* mice have a signalling pathway that alters patterns mimicking human chronic liver diseases. Notably, the CLI‐induced HCC Fah*^−/−^* mouse model is a non‐diabetes‐induced HCC model that serves as a good indicator of HCC in humans.

### 3.2 Metformin prevents hepatocarcinogenesis in Fah^−/−^ mice with CLI

**FIGURE 2:**
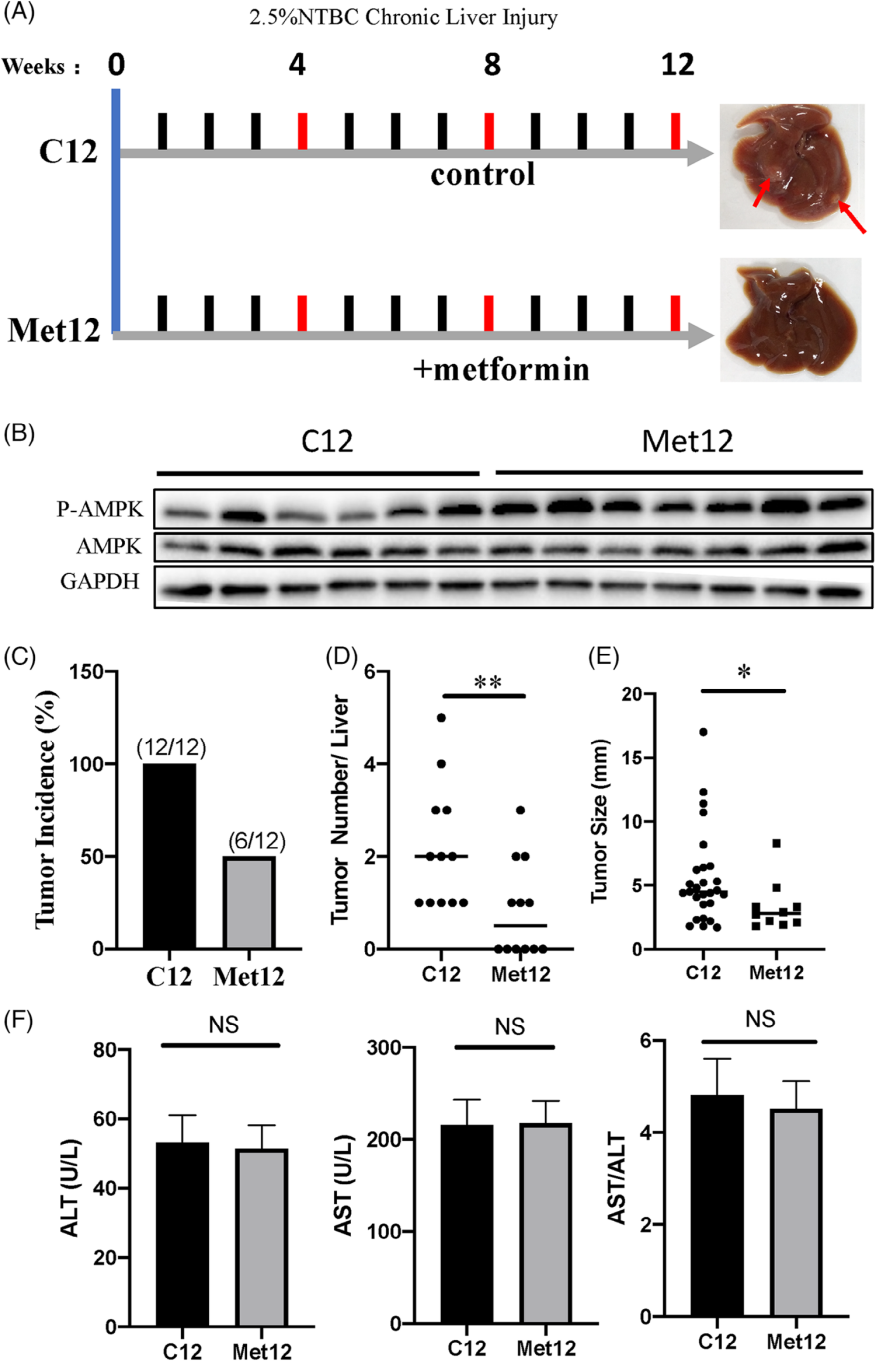
Effects of metformin on the characteristics of precancerous livers and hepatocellular carcinoma (HCC) incidence in Fah^−/−^ mice. (A) Schematic diagram showing the experimental set. Representative photographs of livers with chronic liver injury for 12 weeks without metformin (C12) and with metformin (Met12) are shown. (B) Western blot analysis of P‐adenosine monophosphate‐activated protein kinase (AMPK) and AMPK protein level in liver tissues of C12 and Met12. (C) Graphs representing tumour incidence of Fah^−/−^ mice with and without metformin (Met12 and C12). (D and E) Scatter plots displaying the tumour numbers (D) and size of tumours (E) in Fah‐deficient livers at C12 and with Met12. (F) Serological indexes of liver injury (alanine transaminase [ALT], aspartate transaminase [AST] and AST/ALT) were almost not changed in the serum of Fah^−/−^ mice at C12 and Met12. ^*^ *p* < .05; ^**^ *p* < .01; ^***^ *p* < .001; NS: not significant.

To investigate whether metformin affects HCC formation in CLI Fah*^−/−^* mice (under 2.5% NTBC), the mice were treated with either metformin (Met12) or an equal volume of saline solution (C12) for 12 weeks (Figure 2A). The main pharmacological mechanism of metformin involves the activation of the AMPK pathway.^11^ Western blotting showed that, in our CLI model, AMPK was activated in the metformin‐treated group, indicating the effectiveness of metformin (Figure 2B). HCC was visible in both macroscopic and histological examinations of all Fah*^−/−^* mice (*n* = 12), and metformin treatment significantly delayed tumour formation: only 50% of the metformin‐treated Fah*^−/−^* mice developed HCC after 12 weeks of 2.5% NTBC treatment (*n* = 12) (Figure 2C). Furthermore, Fah*^−/−^* livers without metformin treatment showed significantly more and larger tumours than those treated with metformin (Figure 2D,E). Serum liver function indicators were analysed to confirm the degree of liver injury in both groups. We found that the levels of serum aspartate transaminase (AST), serum alanine transaminase (ALT) and the AST/ALT ratio were not significantly changed in either group (Figure 2F). Taken together, these data indicate that metformin prevents hepatocarcinogenesis in Fah*^−/−^* mice with CLI by activating AMPK signalling.

### 3.3 Metformin suppresses Cyp26a1 expression in hepatocytes in vivo and in vitro

**FIGURE 3:**
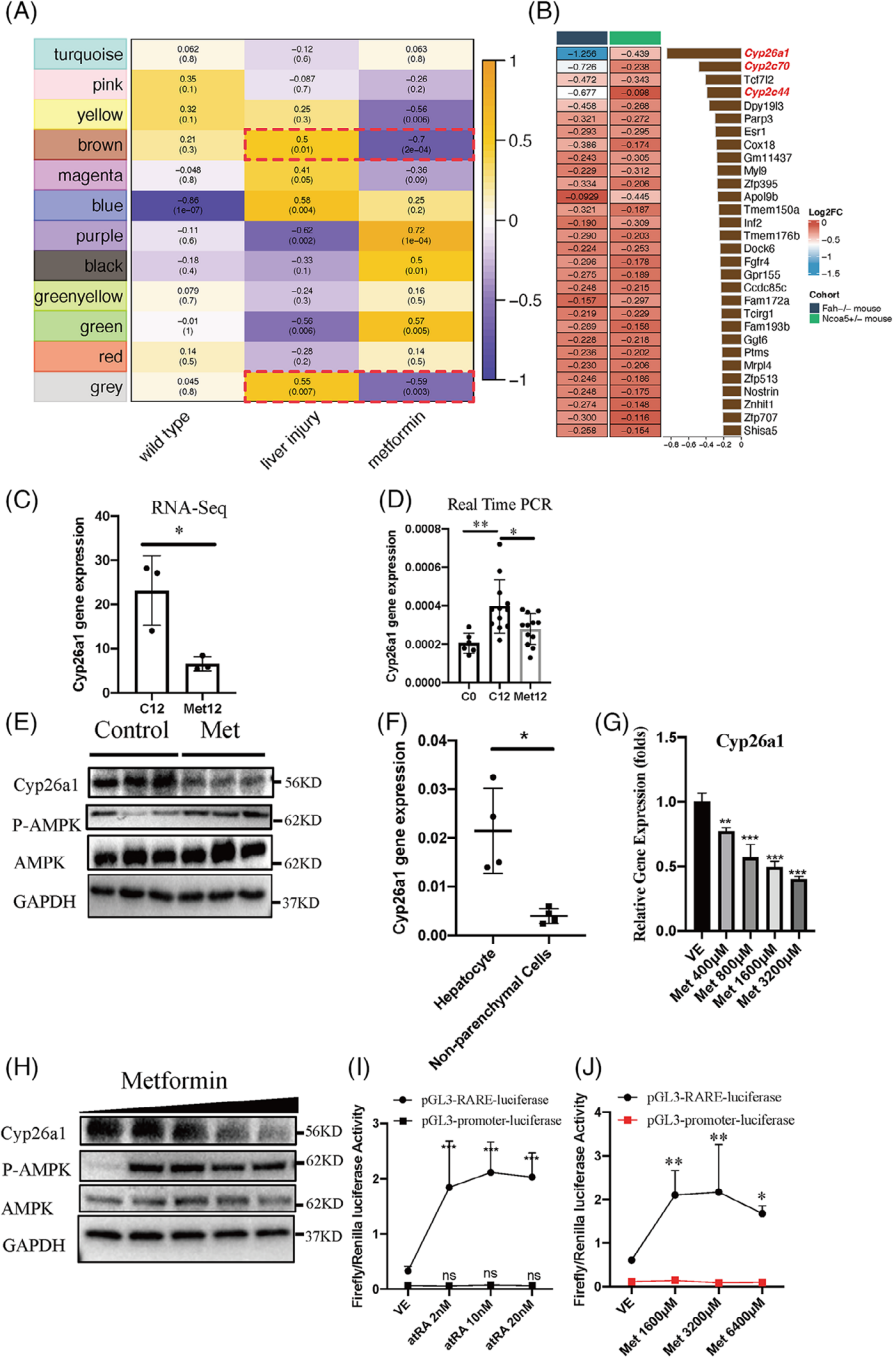
Effects of metformin on Cyp26a1 gene in mouse hepatocytes. (A) Heatmap related to gene modules and traits using weighted gene co‐expression network analysis (WGCNA) pipeline. (B) Fold change of gene expression in Fah^−/−^ and Ncoa5^+/−^ hepatocellular carcinoma (HCC) mouse models with metformin treatment. (C) RNA‐sequencing data of Cyp26a1 gene relative to GAPDH. (D) Quantitative analysis of Cyp26a1 gene in liver tissues from C0, C12 and Met12 was performed by quantitative real‐time polymerase chain reaction (qRT‐PCR) relative to GAPDH. Ordinary one‐way analysis of variance (ANOVA) with Šídák's multiple comparisons test was performed. (E) Western blot analysis of relative protein in liver tissues from C12 and Met12. (F) qRT‐PCR analysis of Cyp26a1 gene expression relative to GAPDH in mouse hepatocytes and non‐parenchymal cells. Perfused hepatocytes and non‐parenchymal cells were separated by low‐speed centrifugation. (G) Perfused hepatocytes treated with metformin in vitro. qRT‐PCR analysis results of Cyp26a1 gene expression relative to GAPDH with different concentrations of metformin. Ordinary one‐way ANOVA with Šídák's multiple comparisons test was performed. (H) Western blot analysis of Cyp26a1 gene expression in perfused adherent hepatocyte with different concentrations of metformin in vitro. (I) Retinoic acid receptor (RARE)‐luciferase assay on Fah^−/−^ hepatocyte cultured with increasing amounts of atRA. Data representative of three independent experiments. Two‐way ANOVA was used. (J) RARE‐luciferase assay on Fah^−/−^ hepatocytes cultured with increasing amounts of metformin. Data are representative of three independent experiments. Two‐way ANOVA was used. Data are represented as the mean ± standard deviation (SD). ^*^ *p* < .05; ^**^ *p* < .01; ^***^ *p* < .001; NS: not significant.

To identify potential AMPK‐targeted genes that mediate the metformin‐induced hindrance of HCC development in Fah*^−/−^* mice, RNA‐seq and corresponding differential gene expression analyses of the livers of Fah*^−/−^* mice with or without metformin treatment were performed. The results revealed that the mRNA levels of 200 genes were significantly altered by metformin treatment (log2‐fold change > 1; Figure S6A and Table S3). From these 200 altered genes, we hypothesised that Cyp26a1 may be the target of metformin in hindering hepatocarcinogenesis. There were several supporting findings, which are listed as follows:

1. Williams et al. have shown that metformin prevents HCC development during CLI in Ncoa5^+/−^ mice.^19^ We comparatively reanalysed our sequencing data with those obtained by Williams et al. (GSE110524). The results revealed 94 common downregulated genes and 107 shared upregulated genes (Figure S6B) in the two mouse models with differently induced HCC. The commonly altered genes were those of signalling pathways during tumourigenesis, including those related to the retinoid metabolic process, retinol dehydrogenase activity, endopeptidase inhibitor activity, lipid metabolic process and glycolysis/gluconeogenesis (Table S4). Therefore, the two mouse models representing differently induced chronic liver injuries may have some common mechanisms that promote the occurrence of HCC. To better realise the similar effects of metformin treatments on HCC induced in both Fah^−/−^ and Ncoa5^+/−^ mouse models, we performed clustering analysis based on the top 1000, 3000 and 5000 Standard Deviation genes after removing the batch effect and using the ‘combat’ function in sva package.^38^ Remarkably, the results showed that the findings for metformin‐treated HCCs induced in Fah^−/−^ and Ncoa5^+/−^ mouse models were much more similar to each other than to the other four groups of wild‐type or untreated controls (Figure S4A). DEGs among all six groups were quantitatively analysed using pairwise comparisons. The results showed that the two metformin‐treated groups had the lowest number of DEGs (Figure S4B). Clustering analysis based on all DEGs also indicated a high similarity between Ncoa5^+/−^ and Fah^−/−^ mice in the metformin‐treated groups (Figure S4C). For validation, we conducted PCA and correlation analyses in all sample groups on the basis of the DEGs in the comparison of metformin‐treated and Fah*^−/−^* non‐treated groups. Consistently, PCA and correlation analyses revealed that the two metformin‐treated groups from the different models were very similar in comparison with the other four groups (wild‐type or non‐treated controls) (Figures S4D and S5A,B). Therefore, metformin treatment may exert a common effect on HCC in both Ncoa5^+/−^ and Fah^−/−^ mouse models.
2. Since a combination analysis of metformin treatment in different mouse models may provide useful information regarding potential therapeutic mechanisms for hepatocarcinogenesis, WGCNA was used to identify the key gene modules for metformin (Figure 3A).^40^ The results showed that the brown and grey gene modules were positively correlated with liver injury traits and negatively correlated with metformin treatment traits. We then collected the significantly active genes in the two modules and investigated their changes in expression in both groups after metformin treatment in comparison with the control groups (Figure 3B). Notably, members of the cytochrome P450 family, including Cyp26a1, *Cyp2c70* and *Cyp2c44*, were downregulated in both metformin treatment groups of Ncoa5*^+/−^* and Fah*^−/^* ^−^ HCC mouse models (Figure 3B).
3. The results also showed that expression of Cyp26a1 was significantly upregulated in C4 in comparison with C0, but was significantly decreased by metformin treatment (Figure 3D). Similar results for Cyp26a1 expression were observed during CLI in Ncoa5^+/−^ mice (Figure S6C). Previous findings also indicated that a decrease in Cyp26a1 led to an increase in atRA, and atRA was demonstrated to be an antitumour metabolite in several tumour types.^50^, ^51^, ^52^, ^53^ Consistently, our results from the GO and KEGG analyses implied that the metabolic signalling pathway of retinol (a precursor of atRA) was enriched during the dynamic process of CLI in Fah^−/−^ mice (Figure 1C, blue arrow), implying that the retinol pathway is essential for HCC formation. We investigated whether decreased Cyp26a1 activity affects HCC formation in CLI Fah^−/−^ mice. We used the Cyp26a1 inhibitor talarozole to inhibit Cyp26a1 activity. Talarozole (R115866) is an oral systemic atRA metabolism‐blocking agent that increases the intracellular levels of endogenous atRA by inhibiting both Cyp26a1 and Cyp26b1.^54^ Fah^−/−^ mice with chronic injury (under 2.5% NTBC) for 12 weeks were treated with oral 2.5 mg/kg talarozole (C12 + Tala4) or solvent alone (C16) for another 4 weeks (Figure S7A). HCC was obviously visible in both macroscopic and histological examinations in all Fah^−/−^ mice. Although the tumour incidence was not significantly different (Figure S7B), talarozole treatment resulted in significantly smaller and fewer tumours in comparison with the control group (Figure S7C,D). As expected, following multiple doses of talarozole, both Cyp26a1 mRNA and protein levels in livers increased significantly. This indicates the autoinduction effect of Cyp26a1 due to increased atRA concentrations and decreased Cyp26a1 activities caused by Talarozole, in line with the previous studies (Figure S7E,F).^55^ All these data demonstrated that inhibition of Cyp26a1 gene expression could inhibit HCC formation in CLI Fah*^−/−^* mice. To better understand therelationship among the CYP26A1 gene, atRA‐related genes and the AMPK‐related pathway in patients with CLI, we utilised ssGSEA for calculating gene set scores and conducted correlation analyses. Intriguingly, we found that CYP26A1 was upregulated in CLI patients (Figure S8A) as well as was negatively correlated with both the atRA‐related genes and AMPK pathway (Figure S8B,C). In parallel, atRA‐related gene set score was downregulated in CLI patients and positively correlated with AMPK pathway (Figure S8D,E). Taken together, the regulatory mechanisms among CYP26A1, AMPK pathway and atRA‐related genes might also exist in clinical patients, similar to Fah^−/−^ mouse model. On the basis of these data, we hypothesised that metformin may suppress the expression of Cyp26a1 to prevent hepatocarcinogenesis in Fah*^−/−^* mice during CLI.
4. RNA‐seq data showed that Cyp26a1 expression was reduced in the metformin‐treated group (Figure 3C). In line with the results of the RNA‐seq analysis, the qRT‐PCR assay indicated that Cyp26a1 mRNA levels were downregulated in the metformin‐treated liver tissues of Fah*^−/−^* mice (Figure 3D). Western blotting also showed that CYP26A1 protein was reduced in metformin‐treated liver tissues (Figure 3E). Cyp26a1 is mostly highly expressed in the liver.^56^ Thus, hepatocytes should also exhibit Cyp26a1 overexpression in the liver of Fah*^−/^* ^−^ mice. The qRT‐PCR results revealed that Cyp26a1 was mainly expressed in purified hepatocytes, but not in non‐parenchymal cells (Figure 3F). The scRNA‐seq data of immune cells from another HBV (Hepatitis B)‐induced HCC model in Fah*^−/−^* mice^42^ also showed that only 23 of the 27 058 immune cells expressed Cyp26a1 (data not shown). Next, we examined whether metformin directly suppresses Cyp26a1 expression in hepatocytes. The perfused hepatocytes were plated onto plates containing Matrigel. Both qRT‐PCR and western blotting showed that the expression of Cyp26a1 in hepatocytes decreased with increasing metformin concentration (Figure 3G,H). CYP26A1 plays a major role in atRA clearance, implying that downregulation of Cyp26a1 leads to elevated atRA levels.^57^ The pGL3‐RARE‐luciferase plasmid contains a retinoic acid receptor (RARE) response element in front of the luciferase (firefly) reporter gene, which reflects the level of atRA.^53^, ^58^ We transfected this plasmid and the control plasmid (pRL‐CMV‐Renilla luciferase) into the primary hepatocytes of Fah*^−/−^* mice with CLI. The firefly/Renilla luciferase activity of pGL3‐RARE‐luciferase increased with atRA treatment while pGL3‐promoter‐luciferase remained unchanged (Figure 3I), indicating that pGL3‐RARE‐luciferase activity could reflect the level of atRA in hepatocytes. We tested the effect of metformin on the pGL3‐RARE‐luciferase and pGL3‐promoter‐luciferase activities, and found that the firefly/Renilla luciferase activity of pGL3‐RARE‐luciferase increased, whereas that of pGL3‐promoter‐luciferase was unchanged with metformin treatment, indicating that metformin increased the level of atRA (Figure 3J). Taken together, these data demonstrate that metformin directly suppresses the expression of Cyp26a1 in hepatocytes, leading to an elevated level of atRA and thereby exerting antitumour effects and preventing tumourigenesis.

### 3.4 Metformin decreases the expression of Cyp26a1 through AMPK/STAT3/Gadd45β/JNK/c‐Jun signalling

**FIGURE 4:**
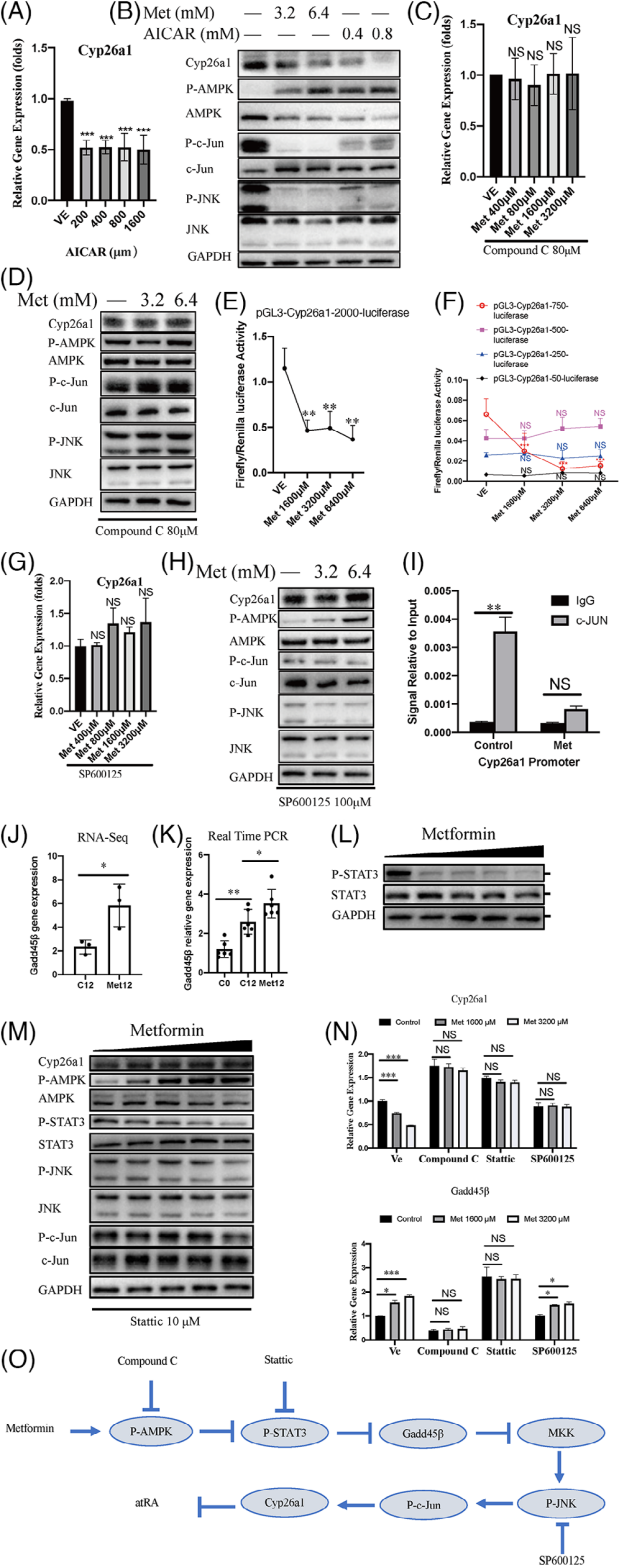
Metformin suppressed Cyp26a1 gene by AMPK/STAT3/Gadd45β/JNK/c‐Jun pathway. (A) Quantitative real‐time polymerase chain reaction (qRT‐PCR) analysis results of Cyp26a1 gene expression in primary hepatocytes from chronic liver injury (CLI) Fah^−/−^ mice treated with different concentrations of 5‐aminoimidazole‐4‐carboxamide1‐β‐D‐ribofuranoside (AICAR). Ordinary one‐way analysis of variance (ANOVA) with Šídák's multiple comparisons test was performed. (B) Western blot analysis of relative gene expression in perfused adherent hepatocyte from CLI Fah^−/−^ mice treated with different concentrations of metformin and AICAR in vitro. (C) qRT‐PCR analysis results of Cyp26a1 gene expression. Primary hepatocytes from CLI Fah^−/−^ mice treated with adenosine monophosphate‐activated protein kinase (AMPK) inhibitor Compound C (80 μM) in the absence or presence of metformin for 6 h. Treatment with the inhibitor started 1 h before metformin treatment. Ordinary one‐way ANOVA with Šídák's multiple comparisons test was performed. (D) Western blot analysis of relative gene expression. Primary hepatocytes from CLI Fah^−/−^ mice treated with AMPK inhibitor in the absence or presence of metformin for 6 h. Treatment with the inhibitor started 1 h before metformin treatment. (E) We transfected plasmid (pGL3‐cyp26a1‐promoter‐2000 bp‐luciferase) into primary hepatocyte from CLI Fah^−/−^ mice cultured with increasing amounts of metformin. Data representative of three independent experiments. Two‐way ANOVA was used. (F) The truncated mouse Cyp26a1 promoter mutation plasmids were separately transfected into primary hepatocyte from CLI Fah^−/−^ mice cultured with increasing amounts of metformin. Data are representative of three independent experiments. Two‐way ANOVA was used. (G) qRT‐PCR analysis results of Cyp26a1 gene expression. Primary hepatocytes from CLI Fah^−/−^ mice treated with JNK inhibitor SP 600125 (100 μM) with increasing amounts of metformin for 6 h. Treatment with the inhibitor started 1 h before metformin treatment. (H) Western blot analysis of relative gene expression. Primary hepatocytes from CLI Fah^−/−^ mice treated with JNK inhibitor SP600125 (100 μM) with increasing amounts of metformin for 6 h. Treatment with the inhibitors started at time of 1 h before metformin treatment. (I) Chromatin immunoprecipitation (ChIP)‐qPCR was carried out after the mouse primary hepatocytes were treated with 4 mM metformin or control using either c‐Jun antibody or immunoglobulin G (IgG) control antibody for immunoprecipitation. (J) RNA‐sequencing (RNA‐seq) data of Gadd45β gene. (K) Quantitative analysis of Gadd45β gene in liver tissues of the treated mice from C0, C12 and Met12 was performed by qRT‐PCR. Ordinary one‐way ANOVA with Šídák's multiple comparisons test was performed. (L) Western blot analysis of relative gene expression in the perfused hepatocytes from CLI Fah^−/−^ mice treated with different concentrations of metformin in vitro. (M) Western blot analysis of relative gene expression. Primary hepatocytes from CLI Fah^−/−^ mice were treated with STAT3 inhibitor stattic (10 μM) with increasing amounts of metformin for 6 h. Treatment with the inhibitors started 1 h before metformin treatment. (N) qRT‐PCR analysis results of Cyp26a1 and Gadd45β gene expression. Primary hepatocytes from CLI Fah^−/−^ mice treated with inhibitor with an increased dose of metformin for 6 h. Treatment with the inhibitor started at the time of 1 h before metformin treatment. Ordinary one‐way ANOVA with Šídák's multiple comparisons test was performed. (O) A diagram shows the possible mechanism by which metformin suppressed Cyp26a1 gene by inhibiting AMPK/STAT3/Gadd45β/JNK/c‐Jun pathway. The experiment was repeated two or three times. Error bars represent standard deviation (SD). ^*^ *p* < .05; ^**^ *p* < .01; ^***^ *p* < .001; NS: not significant.

AMPK is a major effector of metformin, and acadesine (AICAR) is another AMPK activator.^59^ We treated perfused primary adherent hepatocytes of Fah*^−/−^* mice with different concentrations of AICAR. qRT‐PCR and western blotting showed that the expression of Cyp26a1 was also suppressed by AICAR (Figure 4A,B). We speculated that AMPK may be involved in the decreased expression of Cyp26a1 induced by metformin and AICAR. Compound C (dorsomorphin) is an effective, reversible and selective AMPK inhibitor widely used to imply AMPK dependence.^60^ Compound C (80 μM) attenuated the ability of metformin to activate AMPK in mouse hepatocytes (Figure 4D). Both qRT‐PCR and western blotting showed that Compound C attenuated the ability of metformin to suppress the Cyp26a1 expression, indicating that AMPK is a major effector of metformin in suppressing Cyp26a1 gene expression (Figure 4C,D).

To determine whether metformin mediated Cyp26a1 through the repression of its promoter, we constructed the plasmid pGL3‐cyp26a1‐promoter‐2000 bp‐luciferase, which was generated using the promoter of the mouse Cyp26a1 gene (–2000 to +60 bp relative to the transcription start site) to replace the SV40 promoter of pGL3‐promoter‐luciferase. Mouse Cyp26a1 promoter activity was significantly suppressed after metformin treatment (Figure 4E), while pGL3‐promoter‐luciferase activity did not change (Figure 3J), indicating that metformin‐suppressed Cyp26a1 expression was mediated through repression of its promoter.

To define the role of the *cis*‐regulatory elements of the Cyp26a1 promoter in response to metformin, a series of truncated mutants of the Cyp26a1 promoter were generated (–750 to +60 bp, –500 to +60 bp, –250 to +60 bp and –50 to +60 bp). pGL3‐cyp26a1‐promoter‐750 bp‐luciferase also showed significantly decreased luciferase activity following treatment with metformin. The shorter truncated mutants (–500 to +60 bp, –250 to +60 bp and –50 to +60 bp) of the Cyp26a1 promoter blocked metformin‐mediated downregulation of Cyp26a1 promoter activity, indicating that the sequence between nucleotides –750 and –500 bp was critical for the suppression of Cyp26a1 by metformin treatment (Figure 4F). We predicted the transcription factors of this sequence (nucleotides –750 to –500 bp related to the transcription start site) using the PROMO database (http://alggen.lsi.upc.es/cgibin/promo_v3/promo/promoinit.cgi?dirDB = TF_8.3). Forty transcription factors were predicted (Figure S3), and a predicted c‐Jun binding site was located in this region. A previous study showed that the JNK/c‐Jun signalling pathway can be regulated by the metformin–AMPK pathway.^61^ We found that p‐JNK and p‐c‐Jun were inhibited by metformin in hepatocytes (Figure 4B). When AMPK activity was pharmacologically inhibited by Compound C, the metformin‐induced reduction in Cyp26a1, p‐JNK and p‐c‐Jun expression was not observed (Figure 4D). Furthermore, we used the JNK inhibitor SP600125 to determine whether the metformin‐mediated downregulation of Cyp26a1 expression was mediated through the repression of JNK phosphorylation. In the presence of SP600125, JNK phosphorylation was suppressed, and metformin lost its JNK‐inhibiting ability because JNK phosphorylation was inhibited in advance by SP600125. In parallel, Cyp26a1 expression was not decreased by metformin, even when AMPK was activated, and JNK phosphorylation was not suppressed by metformin (Figure 4G,H). The results of the ChIP assay confirmed that c‐Jun directly bound to the Cyp26a1 promoter in primary mouse hepatocytes (Figure 4I). Taken together, these results demonstrated that Cyp26a1 expression was inhibited by metformin through the AMPK/JNK/c‐Jun signalling pathway.

Next, NASH mouse scRNA‐seq data (GSE166504) were used to confirm the regulatory steps from AMPK to JNK/c‐Jun in hepatocytes using SCENIC pipeline for dissecting TF regulatory network from normal tissues (Chow group) to liver‐injured tissues (15, 30 and 34 weeks of NASH). After obtaining and cleaning the scRNA‐seq data with stringent criteria, we obtained various cell types from NASH liver tissues. Particularly, results indicated that Cyp26a1 was mainly expressed by hepatocytes rather than by other cell types (Figure S9A). Then, we conducted sub‐population analysis for hepatocytes and 12 subclusters of hepatocytes were obtained. The results revealed that Cluster 0 was mainly in the Chow group, while Cluster 3 was mainly in the NASH group (15 and 30 weeks) (Figure S9B). In addition, result of AMPK pathway score calculated by ‘addModule’ algorithm in Seurat package showed that the score of Cluster 3 was lower than that of Cluster 0 (Figure S9C). Therefore, AMPK pathway was also gradually inactivated following liver injury, which was consistent with the above result from pseudo‐bulk RNA‐seq of hepatocytes (Figure S9D). Furthermore, SCENIC algorithm was leveraged to predict the TFs of Clusters 0 and 3 (Figure S9E). Remarkably, the Jun‐related TFs, including Jun, Junb and Jund, were all enriched, suggesting a close relationship between AMPK and c‐Jun (Figure S9F,G). The activities of Jun‐related TFs were higher in hepatocytes from NASH mice, consistent with the lower AMPK pathway score (Figure S9D,G). Together, all of the above results confirmed that AMPK pathway was gradually inactivated following liver injury progression and that the AMPK pathway had a close relationship with JNK/c‐Jun in hepatocytes.

To identify potential targets of metformin that induce JNK repression, gene expression differences in the livers of Fah*^−/−^* mice were examined with or without metformin treatment (Figure 3A). We found that the expression of Gadd45β, a JNK inhibitor,^62^, ^63^ was induced in liver tissues after metformin treatment (Figure 4J and Table S3). Expression of Gadd45β in the liver was significantly upregulated after metformin treatment (Figure 4K). Previous studies have shown that JNK activity is inhibited through the metformin‐activated STAT3/Gadd45β/MKK4 pathway during APAP‐induced hepatotoxicity.^64^ Thus, STAT3/Gadd45β/MKK4 pathways were examined in our mouse model. Notably, STAT3 phosphorylation was inhibited by metformin (Figure 4L), which was consistent with previous results showing that metformin inhibits STAT3.^65^, ^66^ In addition, metformin‐induced downregulation of Cyp26a1 expression was abolished by Stattic, the STAT3 inhibitor (Figure 4M). The metformin‐induced downregulation of Cyp26a1 expression was also abolished by AMPK, STAT3 and JNK inhibitors. However, metformin‐induced Gadd45β expression was abolished by both AMPK and STAT3 inhibitors, but not by the JNK inhibitor (Figure 4N). Previous studies have shown that the inhibition of JNK activity during APAP‐induced hepatotoxicity occurs through the metformin‐activated STAT3/Gadd45β/MKK4 pathway.^64^ Together, these results suggested that metformin induced the downregulation of Cyp26a1 expression through precise regulatory effects on each particular member of the AMPK/STAT3/Gadd45β/MKK4 pathways. These effects were involved in the sequential regulatory events from AMPK upregulation to STAT3 downregulation, Gadd45β upregulation, JNK downregulation and c‐Jun downregulation (Figure 4O).

### 3.5 atRA supplementation decreases HCC formation induced by CLI in Fah^−/−^ mice

**FIGURE 5:**
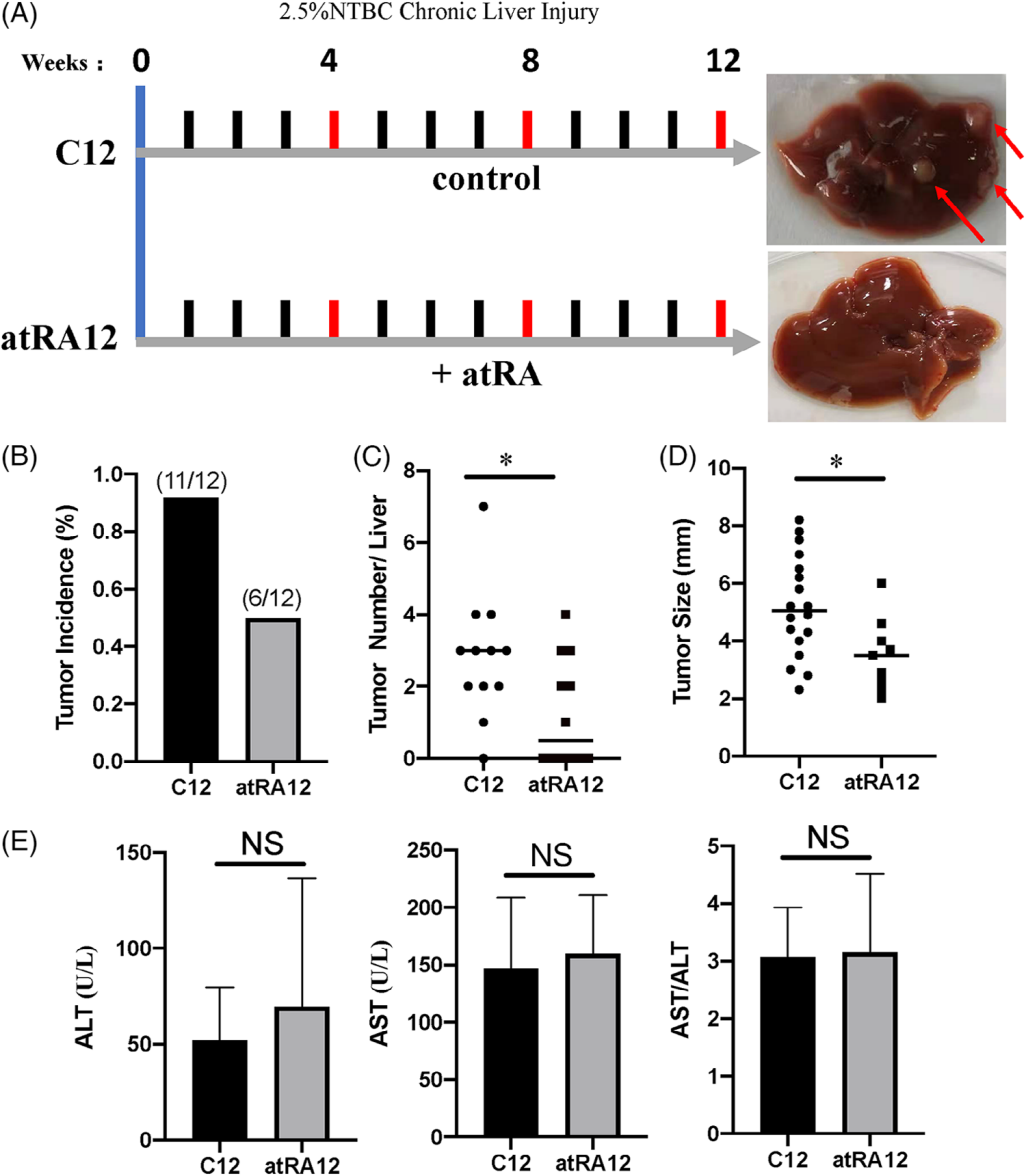
Effects of all‐trans‐retinoic acid (atRA) on hepatocellular carcinoma (HCC) formation in Fah^−/−^ mice with chronic liver injury. (A) Schematic diagram showing the experimental set. Representative photographs of livers with chronic liver injury for 12 weeks without atRA (C12) and with atRA treatment (atRA12) are shown. (B) Graphs representing tumour incidence of Fah^−/−^ mice with and without atRA treatment. (C) Scatter plots displaying the tumour numbers of Fah^−/−^ mice with and without atRA treatment. (D) Scatter plots displaying the tumour size in Fah^−/−^ mice between C12 group and atRA12 group. (E) Indicators of hepatocyte injury (alanine transaminase [ALT], aspartate transaminase [AST] and AST/ALT) were almost not changed in the serum of Fah^−/−^ mice in the C12 and atRA12 groups. ^*^ *p* < .05; ^**^ *p* < .01; ^***^ *p* < .001; NS: not significant.

atRA is widely used for the treatment of cancer and skin, neurodegenerative and autoimmune diseases.^57^ Based on our finding that metformin increased the level of atRA by downregulating Cyp26a1 gene expression (Figure 3), we aimed to determine whether the antitumour effect of atRA could be induced in Fah*^−/−^* mice with CLI. Immunoprecipitation injection of atRA was administered to Fah*^−/−^* mice at the beginning of the induction of CLI (Figure 5A). Tumour incidence, number and diameter were significantly reduced in the atRA‐treated mice in comparison with vehicle‐treated control mice (Figure 5B–D). The levels of serum AST, serum ALT and the AST/ALT ratio did not change significantly in atRA‐treated mice in comparison with controls (Figure 5E). These data demonstrate that atRA exerted antitumour effects in our mouse model.

Cyp26a1 is a hydrolase of atRA, and an increase in atRA levels results in a positive feedback loop that enhances Cyp26a1 expression.^67^, ^68^, ^69^ In the liver and other tissues, atRA‐induced CYP26A1 enzyme metabolism atRA to 4‐hydroxyl‐RA.^70^ To determine whether Cyp26a1 expression was induced or prevented through the changes caused by atRA during hepatocarcinogenesis in Fah*^−/−^* mice, we analysed Cyp26a1 in the livers of Fah*^−/−^* mice after the induction of CLI for up to 12 weeks (named C12) or after induction of CLI plus atRA treatment for up to 12 weeks (named atRA 12). The results indicated that atRA significantly increased Cyp26a1 expression in the liver (Figure S10A). The results also revealed that the atRA‐induced Cyp26a1 expression was dose dependent (Figure S10B).

### 3.6 Both metformin and atRA reduce CD8^+^ T‐cell infiltration and proinflammatory/pro‐tumour cytokines in the liver of CLI Fah^−/−^ mice

**FIGURE 6:**
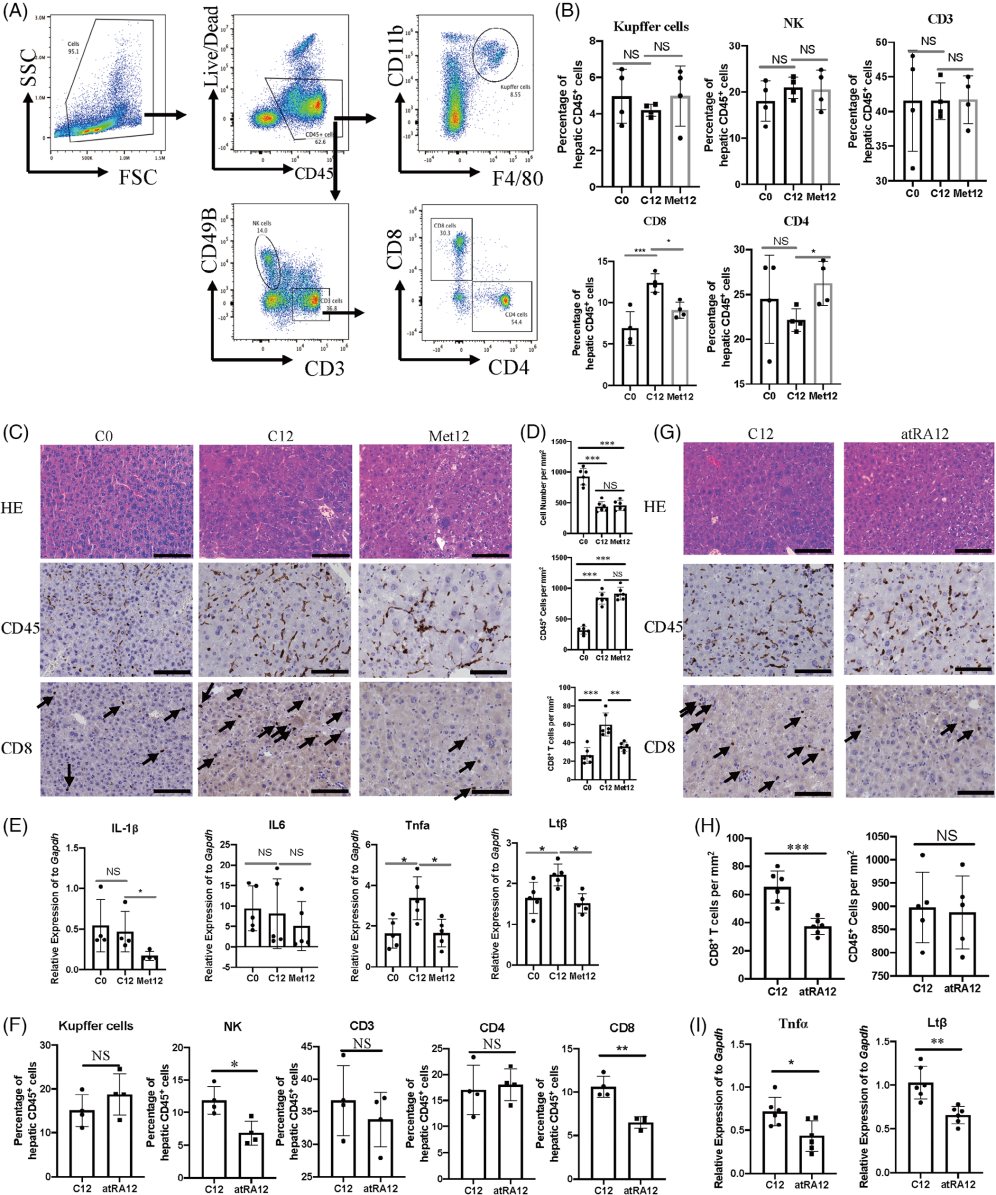
Effects of metformin and all‐trans‐retinoic acid (atRA) on the characteristics of immune cells in Fah^−/−^ mice. (A) Single immune cells were stained with anti‐CD3, CD8, CD45, CD4, CD49B, F4/80, CD11B and live/dead antibodies. The phenotype of liver immune cells was analysed by FACS. (B) Liver‐infiltrating immune cells were measured after treatment with or without metformin. Data represent the mean ± standard deviation (SD) of independent experiments. Statistical significance was determined by ordinary one‐way analysis of variance (ANOVA) with Šídák's multiple comparisons test. (C) Representative pictures of the indicated haematoxylin and eosin (H&E) staining and immunohistochemistry staining. Scale bar, 50 μm. (D) Quantification of hepatocyte cell number, CD45^+^ cell number and CD8^+^ cell number per square millimetre. (E) Quantitative analysis of pro‐tumour cytokines in liver tissues. Ordinary one‐way ANOVA with Šídák's multiple comparisons test was performed. (F) Liver‐infiltrating immune cells were measured after treatment with or without atRA. Data represent the mean ± SD of independent experiments. Statistical significance was determined by ordinary one‐way ANOVA with Šídák's multiple comparisons. (G) Representative pictures of the indicated H&E staining and immunohistochemistry staining. Scale bar, 50 μm. (H) Quantification of CD45^+^ cell number and CD8^+^ cell number per square millimetre in the liver. (I) Quantitative real‐time polymerase chain reaction (qRT‐PCR) analysis of pro‐tumour cytokines in liver tissues. ^*^ *p* < .05; ^**^ *p* < .01; ^***^ *p* < .001; NS: not significant.

Recent studies have shown that metformin can modulate the interaction between tumour cells and their microenvironment, thus presenting an immune‐mediated antitumour effect.^71^, ^72^, ^73^, ^74^ Additionally, the antitumour properties of atRA are closely associated with immune cells.^53^ To explore the mechanisms underlying these tumour‐suppressive effects, we studied the immune cell subsets in CLI Fah*^−/−^* mice treated with metformin or atRA. A prominent increase in the proportion of hepatic CD8^+^ cells among hepatic CD45^+^ cells was found in C12 mice in comparison with C0 mice, and the proportion of CD8^+^ T cells was significantly reduced in metformin‐treated mice (Met12) in comparison with C12 mice, whereas no changes were found in other immune subsets (Kupffer cells, CD4^+^ T, natural killer [NK] and CD3^+^ cells) (Figure 6A,B). Although the proportion of CD4^+^ cells in all CD45^+^ cells was significantly higher in metformin‐treated mice (Met12) than in C12 mice, no difference was observed between C12 and C0 mice. Therefore, we focused on the role of CD8^+^ T cells in the tumour‐inhibiting effects of metformin. Similar results were obtained with IHC staining: the chronically injured liver (C12) contained many more CD45^+^ and CD8^+^ T cells than the liver of mice on 100% NTBC (C12 vs. C0), and the number of CD8^+^ T cells was reduced by metformin treatment, while the number of CD45^+^ cells remained unchanged (Met12 vs. C12) (Figure 6C,D). Accordingly, the number of hepatocytes per field significantly decreased in the histological sections of the CLI Fah*^−/−^* mice in comparison with normal liver tissue sections, which suggested that the size of hepatocytes increased significantly in the CLI Fah*^−/−^* mice in comparison with that in normal livers (C12 vs. C0) (Figure 6D). Hepatic CD8^+^ T cells are integral to antitumour immunity via direct antigen‐specific cytotoxic targeting of tumours. However, recent studies have shown that CD8^+^ T cells contribute to HCC tumour formation in CLI models.^19^, ^25^, ^75^, ^76^ These studies showed that CD8^+^ T cells secrete proinflammatory/pro‐tumour cytokines (interleukin [IL]‐1β, IL‐6, tumour necrosis factor [TNF]‐α and lymphotoxin [Lt]β) in the process of CLI. In particular, CD8^+^ T cells have been demonstrated to be proinflammatory/pro‐tumour immune cells in CLI Fah*^−/−^* mice, although the adopted mouse model is slightly different from our CLI Fah*^−/−^* mice.^25^ We aimed to identify the cytokines that mediate the effect of CD8^+^ T cells on hepatocarcinogenesis in our disease model. The mRNA levels of proinflammatory/pro‐tumour cytokines were measured by qRT‐PCR, and the induction of TNF‐α and Ltβ strikingly correlated with tumour development in the CLI Fah*^−/−^* mice. Moreover, chemokines such as TNF‐α and Ltβ were significantly reduced in metformin treatment (Figure 6E). Taken together, our results indicate that metformin reduces CD8^+^ T‐cell infiltration and proinflammatory/pro‐tumour cytokines to suppress HCC formation in CLI Fah*‐^−/−^* mice.

Next, we sought to elucidate the mechanism underlying the antitumour effects of atRA. We examined CD8^+^ T cells and other immune cells in the atRA‐treated CLI Fah*^−/−^* mice. A significant decrease in the percentage of CD8^+^ T cells was observed after atRA treatment in comparison to that after vehicle treatment (atRA12 vs. C12; Figure 6F). In addition, the percentage of NK cells decreased after atRA treatment but did not change after metformin treatment (Figure 6B). The other immune subsets (Kupffer cells, CD4^+^ T cells and CD3^+^ cells) showed no changes after atRA treatment in comparison to the findings obtained after vehicle treatment. The different effects of metformin and atRA treatment indicated that the signalling pathways regulated by metformin and atRA were not completely consistent in our mouse model. IHC staining also showed that after atRA treatment, the number of CD8^+^ T cells decreased significantly, whereas the number of CD45^+^ cells did not change (Figure 6G,H). We also found that talarozole treatment, which increases the intracellular levels of endogenous atRA, significantly reduced the number of CD8^+^ T cells but did not change the number of CD45^+^ cells (Figure S7G,H). We then tested the expression of proinflammatory/pro‐tumour cytokines secreted by immune cells. qRT‐PCR showed that TNF‐α and Ltβ levels were decreased significantly with atRA treatment in comparison with those in untreated cells, similar to metformin treatment (Figure 6I). These data indicate that the inhibition of HCC by metformin was at least partly due to the promotion of elevated atRA levels.

### 3.7 Hepatic resident‐like CD8^+^ T cells increase gradually following the development of CLI in Fah^−/−^ mice

**FIGURE 7:**
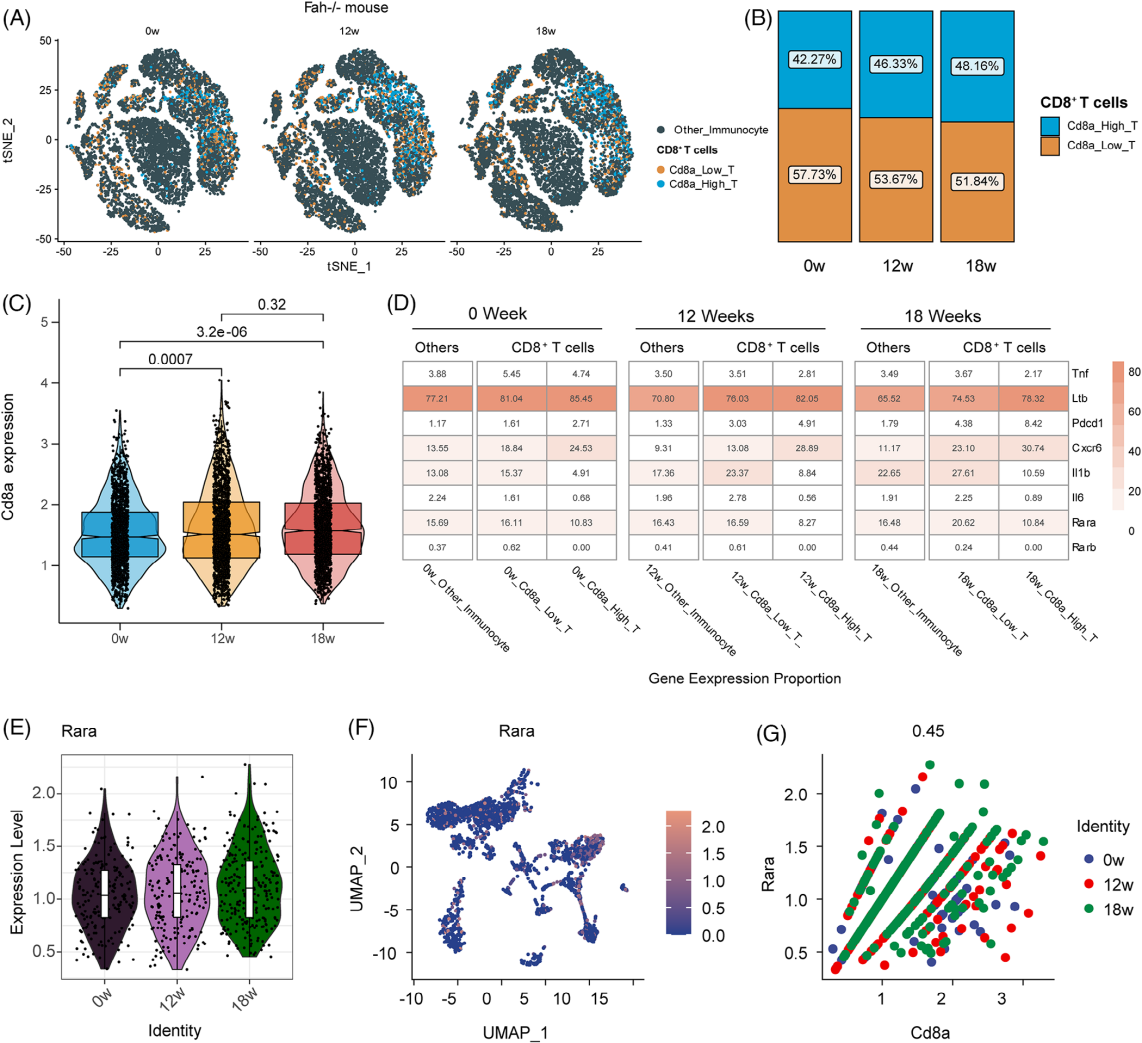
Single‐cell RNA‐sequencing analysis reveals the expression characteristics of CD8^+^ T cells in Fah^−/−^ mouse with progressive chronic liver injury. (A) T‐distributed stochastic neighbour embedding (t‐SNE) plot of immune cells of Fah^−/−^ mouse at 0, 12 and 18 weeks under chronic liver injury (CLI). (B) Histogram indicating the proportion of CD8^+^ T cells at 0, 12 and 18 weeks. (C) Violin plot presenting the expression levels of Cd8a at 0, 12 and 18 weeks. (D) Expression proportion of Tnf, Ltb, Pdcd1, Cxcr6, Il1b, Il6, Rara and Rarb in CD8^+^ T cells and other immune type groups at 0, 12 and 18 weeks. (E) Violin plot presenting the expression levels of Rara at 0, 12 and 18 weeks. (F) Uniform manifold approximation and projection (UMAP) plot showing the expression levels of Rara. (G) Correlation analysis of Cd8a and Rara in Fah^−/−^ mouse at 0 week (blue), 12 weeks (red) and 18 weeks (green).

To determine whether CD8^+^ T cells express proinflammatory/pro‐tumour cytokines and the atRA receptor, we obtained related scRNA‐seq data. To confirm the consistency of transcriptome levels with the conclusions obtained using flow cytometry assays, CD8^+^ T cells were divided into a Cd8a‐high‐expression group and a Cd8a‐low‐expression group to better observe the dynamic changes in CD8^+^ T cells during tumour progression at the mRNA level. CD8^+^ T cells with high Cd8a expression were more likely to be in the same cluster, whereas CD8^+^ T cells with low Cd8a expression were distributed separately and mixed with other immune cells (Figure 7A). Interestingly, the results showed that the number of CD8^+^ T cells, especially CD8^+^ T cells with high Cd8a expression, gradually increased after CLI (Figures 7A–C and S11A–C). We found that the proportion of CD8^+^ T cells expressing proinflammatory/pro‐tumour cytokines/molecules was higher than that of other immune cells, especially Ltb, Pdcd1, Cxcr6 and Il1b (Figure 7D). The expression levels of most pro‐tumour cytokines were higher in CD8^+^ T cells with high Cd8a expression (Figure S11D). In addition, the expression levels of the atRA receptors (Rara and Rarb) gradually increased following CLI (Figures 7E and S11E). However, CD8^+^ T cells tended to express Rara, whereas few cells expressed Rarb (Figures 7F and S11F). The expression levels of both Rara and Rarb correlated with the levels of CD8^+^ T cells (Figures 7G and S11G). We further explored the expression characteristics of proinflammatory/pro‐tumour molecules in CD8^+^ T cells. Intriguingly, Tnf, Pdcd1, Cxcr6, Il1b and Il6, excluding Ltb, were all gradually upregulated along the tumourigenesis of Fah*^−/−^* mouse model (Figures S11–S, 17A). Tnf, Ltb, Pdcd1, Il1b and Cxcr6, but not Il6, were highly expressed and distributed in the same CD8^+^ T‐cell clusters (Figures S11–S, 17B). All these pro‐tumour genes were correlated with the expression of Cd8a (Figures S11–S, 17C) and Rara (Figures S11–S, 17D). In short, CD8^+^ T cells were responsible for the liver tumourigenesis of Fah^−/−^ mice via expressing pro‐tumour molecules.

To further characterise whether the proinflammatory/pro‐tumour CD8^+^ T signature (Cd8a, Tnf, Ltb, Pdcd1, Il1b, Il6 and Cxcr6) existed in other mouse models of CLI, the Addmodule algorithm was also used to calculate the activity score of the proinflammatory/pro‐tumour CD8^+^ T signature in NASH mice using the online open‐access scRNA‐seq data (Figure S18A–C). The results indicated that the proinflammatory/pro‐tumour CD8^+^ T signature was gradually and consecutively activated following the induction of liver injury from chow at 15, 30 and 34 weeks of treatment (Figure S18B,D), indicating that the worse the liver injury, the higher the score of the CD8^+^ T signature. In this study, scRNA‐seq analysis revealed that proinflammatory/pro‐tumour CD8^+^ T cells are involved in liver tumourigenesis in CLI mice.

### 3.8 Hepatic resident‐like CD8^+^ T cells were upregulated in patients with CLI

**FIGURE 8:**
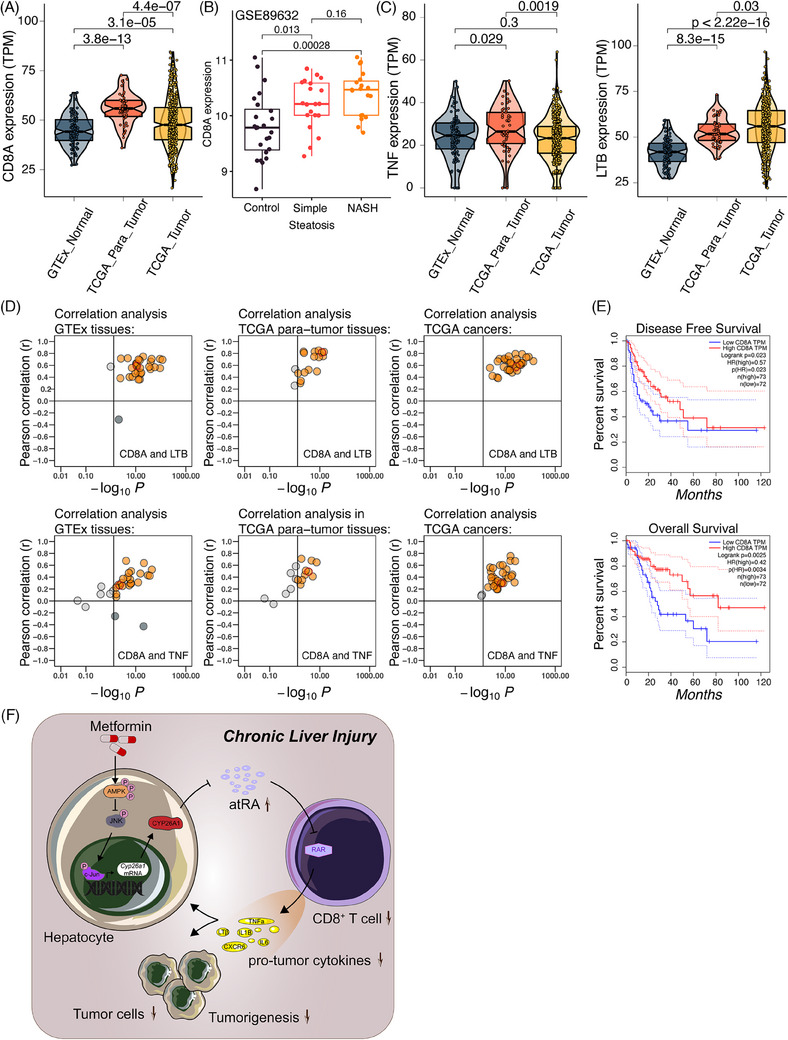
CD8^+^ T cells have different roles in hepatocellular carcinoma (HCC) and HCC‐adjacent tumour based on their molecular and functional characteristics. (A) Violin plot presenting the expression levels of CD8A in GTEx normal, The Cancer Genome Atlas (TCGA) para‐tumour and TCGA tumours tissues. (B) Boxplot presenting the expression levels of CD8A in GSE89632 healthy control (HC), simple steatosis (SS) and non‐alcoholic steatosis hepatitis (NASH) tissues. (C) Violin plot presenting the expression levels of tumour necrosis factor (TNF) and LTβ in GTEx normal, TCGA para‐tumour and TCGA tumours tissues. (D) Correlation analysis of CD8A and LTβ or TNF in 30 GTEx normal, 17 TCGA para‐tumour and 34 TCGA tumour tissue types. Red colour indicates the liver organ in GTEx or HCC para‐tumour tissues, and HCC tumour in TCGA tumour tissues. (E) Kaplan–Meier survival analysis of overall survival (OS) (left) or disease‐free survival (DFS) for CD8A transcript levels in HCC patients from the TCGA database. (F) Schematic diagram illustrating the mechanism of appropriately metformin‐downregulated tumour promotion in hepatocarcinogenesis. The icons of cell types were obtained from SERVIER MEDICAL ART (https://smart.servier.com/).

To assess the relevance of our findings between precancerous mouse livers and human HCCs, adjacent liver tissues and HCC samples from TCGA and normal liver tissues from the GTEx database were downloaded and analysed for the expression levels of CD8A and proinflammatory/pro‐tumour cytokines. We found that CD8^+^ T cells increased in the adjacent tumour tissues compared to both HCC and normal liver tissues (Figure 8A). Comparisons among HC, SS and NASH samples from the GSE89632 database showed an increase in the number of CD8^+^ T cells in the chronically injured human liver (Figure 8B). Notably, a previous study also found that the fraction of CD8^+^ T cells was higher in HCC and HCC‐adjacent tissues than in healthy liver tissues, whereas HCC‐adjacent tissues contained even more T cells than HCC tissues,^77^ in agreement with the findings of our analysis (Figure 8A). We also found that all pro‐tumour cytokines were upregulated in tumour‐adjacent tissues, whereas they were not consistently upregulated in tumour tissues in comparison with normal tissues (Figures 8C,D and S19). Moreover, we found a significantly higher positive regression coefficient between CD8A expression and TNF‐α or LTβ in adjacent tumour tissues than in HCC tissues and normal tissues (Figures 8D and S20–S22A). In parallel, the other four pro‐tumour molecules showed a consistent pattern similar to TNF‐α or LTβ (Figures S20–S22B). Pan‐tissue and pan‐cancer analyses showed a significant correlation between CD8^+^ T cells and these pro‐tumour cytokines in most tissues and cancer types, especially in the liver tissues and HCC, suggesting that CD8^+^ T cells contribute to the expression of pro‐tumour cytokines (Figures 8D and S20–S22B). Taken together, these results indicate that CD8^+^ T cells express proinflammatory/pro‐tumour cytokines and may have different expression levels and functions in adjacent tumour and HCC tissues.

Next, we examined the clinical significance of CD8A expression in liver cancer. When patients were divided into ‘high’ and ‘low’ CD8 expression based on the top 20% value of CD8A, we observed that elevated CD8A expression was associated with better patient survival in the TCGA liver cancer database (Figure 8E). This association suggests that CD8^+^ T cells may function as pro‐tumour cells in adjacent tumour tissues and as antitumour cells in tumour tissues, depending on the expression levels of pro‐tumour or antitumour cytokines secreted by the CD8^+^ T cells. Altogether, these results show that the number and function of hepatic resident‐like CD8^+^ T cells are altered in livers with varying degrees of injury (normal liver, chronically injured liver and HCC).

Taken together, these results showed that metformin suppressed the expression of Cyp26a1 through the AMPK/JNK/c‐Jun pathway, leading to elevated atRA levels. Moreover, atRA treatment inhibited CD8^+^ T‐cell proliferation by binding to the atRA receptor (RAR) of CD8^+^ T cells.^78^, ^79^ The decrease in CD8^+^ T‐cell infiltration can reduce the release of proinflammatory/pro‐tumour cytokines (especially TNF‐α and Ltβ) and thereby inhibit hepatocarcinogenesis (Figure 8F).

## 4 DISCUSSION

The existing systemic options for the prevention and treatment of HCC are limited, indicating an urgent need for new therapeutic targets, preventive strategies and biomarkers for patient stratification. Thus, evaluation of new therapeutic targets and preventive strategies using animal models is expected to be of potential clinical value. HCC development is a complex, multistep process caused by diverse risk factors, indicating that no adaptive animal model can fully mimic the occurrence and development of HCC in humans. Fah*^−/−^*‐induced HCC shows a very low incidence rate in mice, and CLI Fah^−/−^ mice are a suitable animal model that represents most phenotypic and biochemical characteristics of patients with FAH deficiency.^24^ Furthermore, the HCC model of CLI‐induced tumour in Fah*^−/−^* mice is similar to human alcohol‐induced HCC or c‐myc‐altered HCC.^25^ Many studies have used this mouse model to discover and verify relevant biological phenomena and mechanisms related to HCC. These results indicated that HCC induced in Fah‐deficient mice with CLI could be a good indicator of human HCC.

In this study, we observed altered transcriptomic profiles and signalling pathways in the early stages of CLI during hepatocarcinogenesis, and identified hepatic responses to metformin treatment. Our results revealed that the inhibition of AMPK activity and symptoms of hypoglycaemia coexisted in the CLI model (Figure 1D,F). In addition, AMPK activity is repressed in various human liver diseases (Figure 1G–I).^46,47^ AMPK activity is reduced by inflammation, obesity and diabetes, and activation of the AMPK pathway has been viewed as a viable therapeutic strategy to improve HCC.^80^ These results suggest that metformin may improve various liver diseases by upregulating repressed AMPK activity under disease conditions.

Metformin is an AMPK activator and blood glucose regulator, which prompted us to investigate the role and mechanism of action of metformin in our non‐diabetic HCC model. Epidemiological studies have shown a reduction in the incidence of and mortality from liver cancer in patients with type 2 diabetes treated with metformin.^81^, ^82^, ^83^ In our mouse model, metformin treatment significantly reduced the HCC incidence in Fah*^−/−^* mice (Figure 2C–E). Chronic inflammation contributes to hepatocarcinogenesis through multiple mechanisms, including the secretion of proinflammatory and pro‐tumourigenic cytokines by immune cells. The number and proportion of CD8^+^ T cells increased significantly during CLI, and metformin could reverse this effect in Fah*^−/−^* mice (Figure 6B–D). CD8^+^ T cells can be classified on the basis of their molecular and functional characteristics. Generally, tumour‐infiltrating CD8^+^ T cells are antitumour immune cells associated with favourable prognosis.^84^, ^85^ On the other hand, CD8^+^ T cells showing a high expression of inhibitory receptors (PD‐1, TIGIT, TIM‐3, LAG‐3) are defined as exhausted CD8^+^ T cells, which progressively lose effector function and indicate a poor outcome.^86^, ^87^

Several recent studies have shown that CD8^+^ T cells contribute to the development of HCC in several types of chronic liver injuries in mice.^19^, ^25^, ^75^, ^76^ Endig et al. showed that CD8^+^ T cells and LTβ signalling contribute to HCC development in Fah*^−/−^* mice with CLI, which is exactly the same as the mouse model we used; however, their method of inducing CLI was slightly different.^25^ Pfister et al. reported that in a preclinical model of NASH‐induced HCC, CD8^+^ T cells contributed to the induction of NASH‐HCC rather than invigorating or executing immune surveillance.^76^ Concordantly, another group revealed that autoaggression of CD8^+^ T cells in the liver may be involved in the development of HCC in patients with NASH.^75^ Williams et al. also observed the enrichment of exhausted CD8^+^ T cells in the precancerous liver of Ncoa5^+/−^ mice and reported that these T cells function as a proinflammatory and pro‐tumourigenic microenvironment. Metformin treatment can reduce hepatic infiltration of CD8^+^ T cells and limit HCC formation in CLI in Ncoa5^+/−^ mice.^19^ These findings suggest that the classification (effector or exhaustion) and location (tumour or adjacent tumour) of CD8^+^ T cells determines whether they are pro‐tumour or antitumour T cells. Our data showed that the number and ratio of CD8^+^ T cells increased during CLI, and metformin inhibited HCC by reducing CD8^+^ T‐cell infiltration and proinflammatory/pro‐tumour cytokines secreted by CD8^+^ T cells in the livers of CLI Fah*^−/−^* mice (Figure 6B–D). scRNA‐seq analysis of CD8^+^ T cells in Fah*^−/−^* mice revealed that CD8^+^ T cells express proinflammatory/pro‐tumour cytokines and atRA receptors (Figure 7). Interestingly, this study also found that the fraction of CD8^+^ T cells was higher in HCC and HCC‐adjacent tissues than in healthy liver tissues, whereas HCC‐adjacent tissues contained more CD8^+^ T cells than HCC (Figure 8).^77^ Thus, in addition to providing insights into the mechanism of action of metformin, our data could be valuable in supporting the idea that metformin treatment may be beneficial in reversing the CD8^+^ T‐cell‐exhausted tumour microenvironment in patients with HCC.

RA is an active metabolite of vitamin A and includes atRA, 9‐cis‐retinoic acid (9‐cis RA) and 13‐cis‐retinoic acid (13‐cis RA).^57^ Vitamin A is irreversibly converted into atRA by aldehyde dehydrogenase (ALDH) family members. atRA is degraded by atRA‐degrading cytochrome P450 reductases, such as Cyp26a1, which convert atRA to inactive metabolites.^57^ Both the upregulation of ALDH and downregulation of Cyp26a1 lead to an increase in atRA levels. In our study, the expression of Cyp26a1*‐* was decreased by metformin treatment in vivo and in vitro (Figure 3). Decreased Cyp26a1 expression results in increased atRA levels. In addition, metformin directly increased the luciferase activity of the atRA response element plasmid, further demonstrating that metformin promotes atRA levels in hepatocytes (Figure 3J). An increasing number of preclinical and clinical studies have attempted to evaluate the efficacy of atRA against tumours. A classic example of its clinical use is in the treatment of acute promyelocytic leukaemia, which represents the most effective use of atRA in cancer therapy.^50^, ^51^ Bryan et al. reported that atRA combined with paclitaxel showed better overall clinical efficacy than paclitaxel alone in the treatment of recurrent or metastatic breast cancer.^52^ Han et al. showed that retinaldehyde storage was significantly decreased in patients with HCC and found that retinol metabolism has great potential for clinical application in the diagnosis, prognosis and chemotherapy of HCC.^88^ Several mechanisms are related to the antitumour activity of atRA, including its effect on the immune system.^53^, ^78^ In our study, we also demonstrated that atRA agents possess antitumour activity through their ability to reduce the number and ratio of CD8^+^ T cells in Fah*^−/−^* mice (Figure 6). CD8^+^ T cells express the atRA receptor RAR (Figures 7 and S8D) and respond to atRA.^89^ Treatment with atRA inhibits T‐cell proliferation in a dose‐dependent manner in vivo and in vitro.^78^, ^79^ These results suggest that metformin may have reduced the number and ratio of CD8^+^ T cells by increasing atRA levels in our mouse model. This is the first time that the traditional clinical drug metformin has been associated with the metabolite atRA. The metformin/Cyp26a1/atRA axis may partly explain the effects of metformin on CD8^+^ T‐cell proliferation and differentiation. Because many signalling pathways have been reported to be involved in the antitumour roles of metformin, we do not rule out other cancer suppression pathways for metformin in our model, and thus, other potential mechanisms are worth further exploration. Through the exploration of the new mechanism of metformin in present work, we can screen out some drugs that can be used together with metformin, thereby achieving better therapeutic effects in clinical practice and promoting the application of metformin in treating and preventing HCC.

## 5 CONCLUSIONS

Both metformin and atRA were antineoplastic agents. Metformin and atRA reduced CD8^+^ T‐cell infiltration and proinflammatory/pro‐tumour cytokines secreted by CD8^+^ T cells in the formation of HCC during CLI. In summary, this study is the first to show that metformin increases the level of atRA by inhibiting the expression of Cyp26a1 and inhibiting HCC caused by CLI.

## AUTHOR CONTRIBUTIONS

WH, MC, CL designed and performed experiments, analyzed data; WC, YY, YC, ZY, KM, MX performed animal experiments; XW, GW, LP, ZD performed bioinformatics analysis; WH, YC, JW performed immunohistochemistry analysis; ZH conceptualized study, supervised and planned research. WH, XW, ZH wrote the paper.

## DECLARATION OF COMPETING INTEREST

The authors declare no potential conflicts of interest.

## CONFLICT OF INTEREST STATEMENT

The authors declare they have no conflicts of interest.

## ETHICS STATEMENT

Patient specimens were obtained from the sample information service platform of Shanghai East Hospital under protocols approved by Shanghai East Hospital. The study protocol conformed to the ethical guidelines approved by the Shanghai East Hospital Ethics Committee, and written informed consent was obtained from each patient.

## Supporting information

Supporting Information

Supporting Information

Supporting Information

Supporting Information

Supporting Information

## Data Availability

The raw RNA sequence data were generated by OE Biotech Co., Ltd. All data generated or analysed during this study are included in the manuscript and supporting files, and the source data files have been provided in Figures 1 and 3.
