# Supplementary material for: Metformin reduces hepatocarcinogenesis by inducing downregulation of Cyp26a1 and CD8+ T cells
Source: Clin Transl Med. 2023 Nov 23;13(11):e1465. doi: 10.1002/ctm2.1465 (PMC10668005; doi:10.1002/ctm2.1465)
Supplement: Supplementary file 1 — Supporting Information [file CTM2-13-e1465-s001.docx]

**
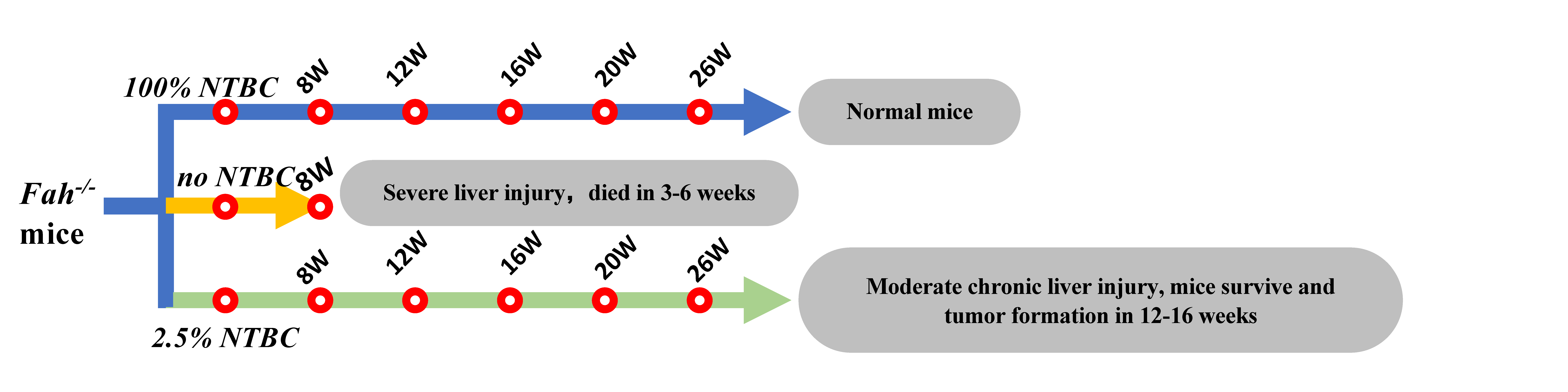
**

**Figure S1. characteristics of *Fah*^-/-^ mice.** under 100% NTBC, the liver of Fah^-/-^ mice was preserved normally; Fah^-/-^ mice developed acute liver injury and died at 3-6 weeks without any NTBC; Fah^-/-^ mouse can survive under 2.5% NTBC, but experience chronic liver injury and form liver cancer after 12 weeks.





**Figure S2. AMPK pathway was suppressed in chronic liver injury tissues compare with normal tissues.**(A)Heatmap of AMPK pathway and related genes in normal andpremalignant tissues. (B)Expression level of AMPK pathway related genes in normal and premalignant tissues (GSE148355 dataset). (C) Heatmap of AMPK pathway and related genes in normal tissues and NAFLD tissues. (D) Expression level of AMPK pathway related genes in normal tissues and NAFLD tissues (GSE89632 dataset). Normal: non-tumor Normal control, FL: low Fibrosis, FH: high Fibrosis, CS: Cirrhosis (CS), DL: Dysplastic nodule Low, DH: Dysplastic Nodule high, NASH: nonalcoholic steatohepatitis


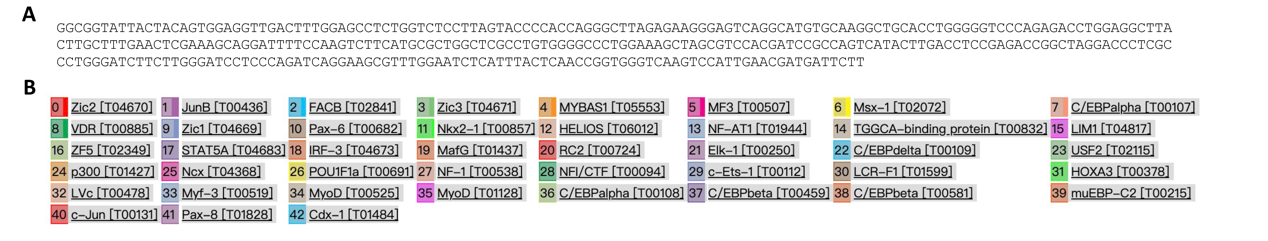


**Figure S3. Predict transcription factors that bind in the promoter region of nucleotide -750 to -500 relation to transcription start site of mouse cyp26a1 gene.**

(A)The detail sequence of nucleotide -750 to -500 relation to transcription start site of mouse cyp26a1 gene.

(B) All the predicted transcription factors binding to nucleotide -750 to -500 relation to transcription start site of mouse cyp26a1 gene using PROMO database (http://alggen.lsi.upc.es/cgibin/promo_v3/promo/promoinit.cgi?dirDB=TF_8.3)


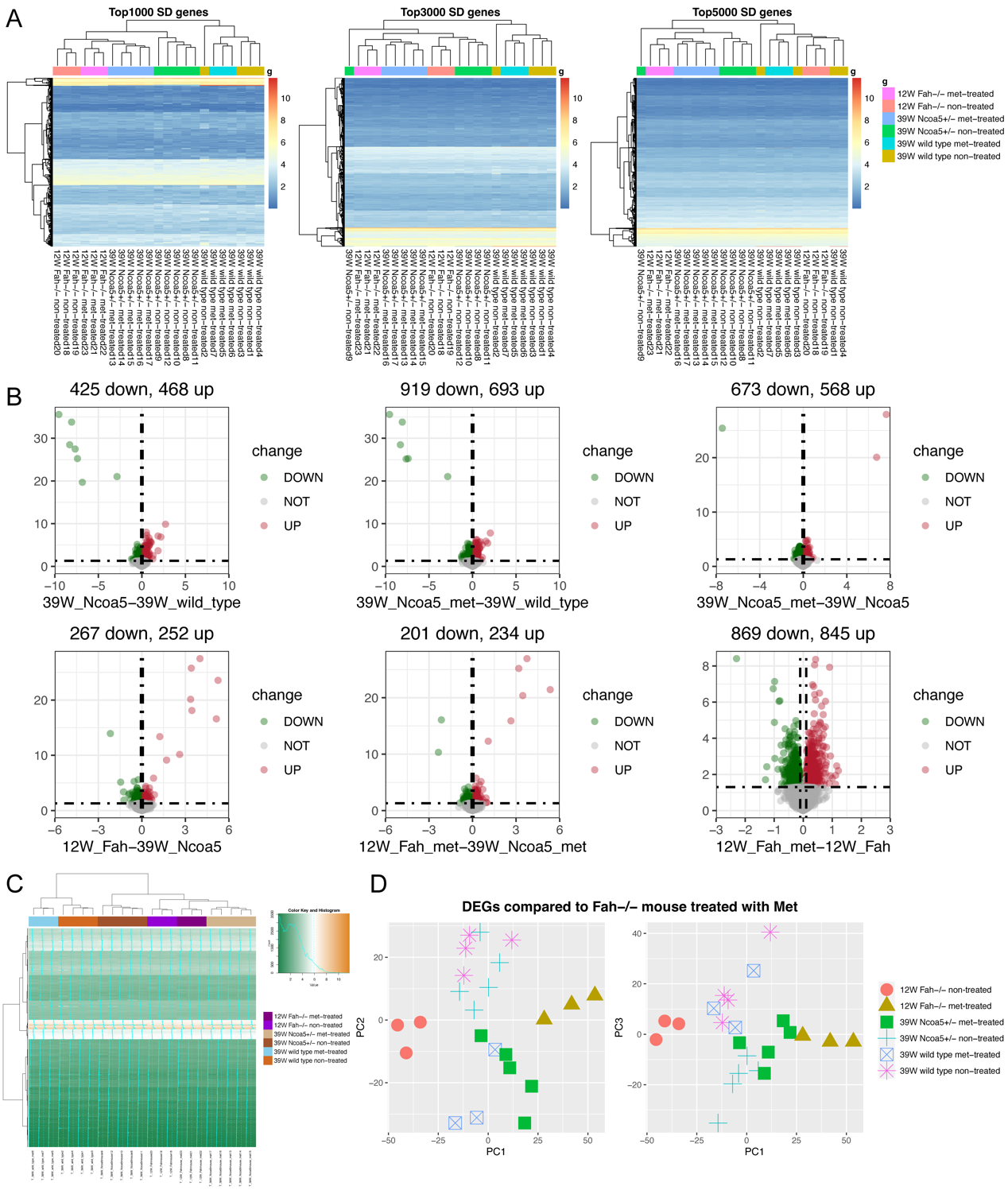


**Figure S4. Transcriptomic comparison of** ***Fah*^-/-^ and *Ncoa5*^+/-^ HCC mouse models.** (A) Heatmap and clustering analysis of all samples from *Fah*^-/-^ and *Ncoa5*^+/-^ HCC mouse models. SD indicates Standard Deviance. (B) Volcano plot displays the total numbers of differentially expressed genes between each two groups. (C) Heatmap and clustering analysis of all samples from *Fah*^-/-^ and *Ncoa5*^+/-^ HCC mouse models based upon all DEGs. (D) PCA of all samples from *Fah*^-/-^ and *Ncoa5*^+/-^ HCC mouse models based on DEGs compared to *Fah*^-/-^ HCC mouse model treated with metformin.


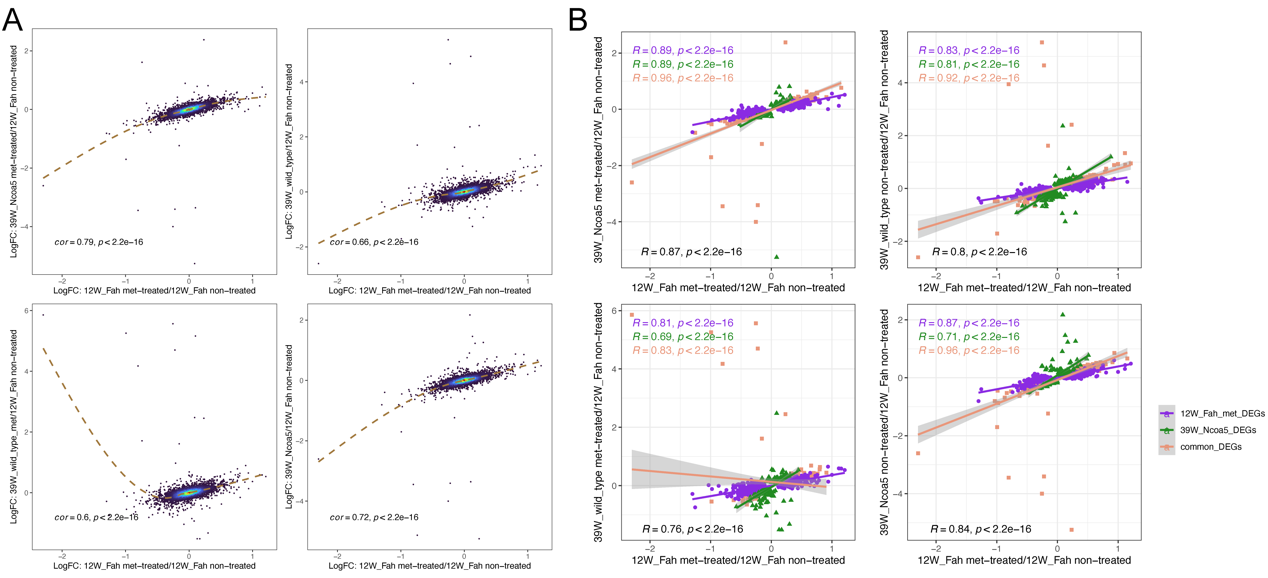


**Figure S5. Correlation analysis of different groups in** ***Fah*^-/-^ and *Ncoa5*^+/-^ HCC mouse models.** (A) Correlation analysis of all genes between the logFC of “12W *Fah*^-/-^ met-treated/12W *Fah*^-/-^ non-treated” to all other four groups, respectively. FC represents “Fold Change”. All genes were showed as points. (B) Correlation analysis of DEGs between the logFC of “12W *Fah*^-/-^ met-treated/12W *Fah*^-/-^ non-treated” to all other four groups, respectively. DEGs were showed as points. Pink color means the common DEGs; Purple color indicates “12W *Fah*^-/-^ met-treated/12W *Fah*^-/-^ non-treated” DEGs; Green color represents other four *Ncoa5*^+/-^ HCC mouse model- related DEGs compared to different controls.
