# Supplementary material for: Metformin reduces hepatocarcinogenesis by inducing downregulation of Cyp26a1 and CD8+ T cells
Source: Clin Transl Med. 2023 Nov 23;13(11):e1465. doi: 10.1002/ctm2.1465 (PMC10668005; doi:10.1002/ctm2.1465)
Supplement: Supplementary file 2 — Supporting Information [file CTM2-13-e1465-s003.docx]

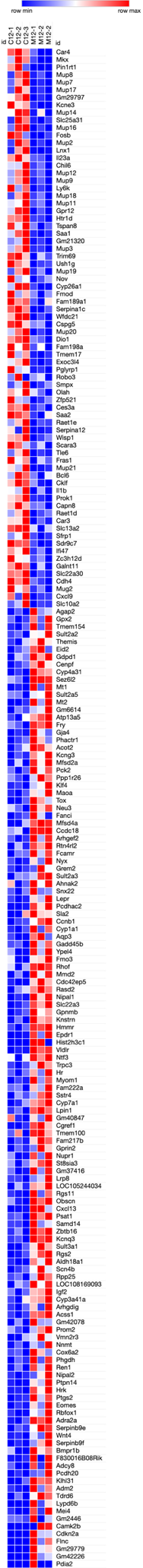

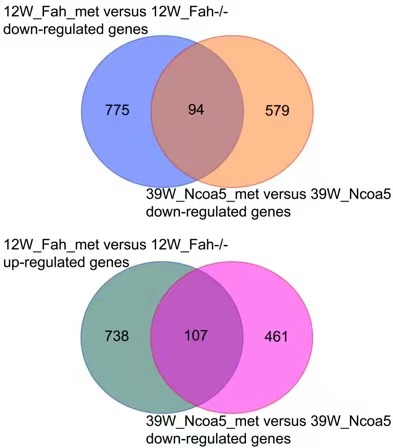


**Cyp26a1**

**A**

**B**

**C**

**Figure S6.** **Effects of metformin on *Cyp26a1* Gene Expression.**

**(**A) Differentially up- or down- regulated genes between C12 and Met12. (Log2 Fold Change >1, FDR<0.05). (B) The intersection of our sequencing results and GSE110524. After removing the batch effect, there are 94 common down-regulated genes and 107 shared up-regulated genes. (c) Expression levels of Cyp26a1 in liver tissues of WT, WT with metformin, Ncoa5+/- and Ncoa5+/- with metformin treatment in GSE110524 database.

**Figure S7.** **Effects of Cyp26a1 inhibitor on the characteristics of precancerous livers and HCC incidence in *Fah^-/-^* mice**

(A) Schematic diagram showing the experimental set. Representative photographs of livers about chronic liver injury for 16 weeks without Talarozole (C16) and with Talarozole for 4 weeks (C12+Tala4) are showing. n=6, each group. (B) Graphs representing tumor incidence of *Fah^-/-^* mice with and without Talarozole. (C-D) Scatter plots displaying the tumor numbers (C) and size of tumors (D) in Fah-deficient livers. (E) Quantitative analysis of *Cyp26a1* gene in liver tissues from C16 and C12+Tala4 was performed by qRT-PCR relative to *Gapdh*. (F) Western blot analysis of Cyp26a1 protein level in liver tissues of C16 and C12+Tala4. (G) Representative pictures of the indicated H&E staining and immunohistochemistry staining. Scale bar, 50μm. (H) Quantification of CD45^+^ cell number and CD8^+^ cell number per square millimeter in the liver. Data represent “mean ± SD” for independent experiments. Statistical significance was determined by unpaired two-tail t-test. (***P < 0.001).


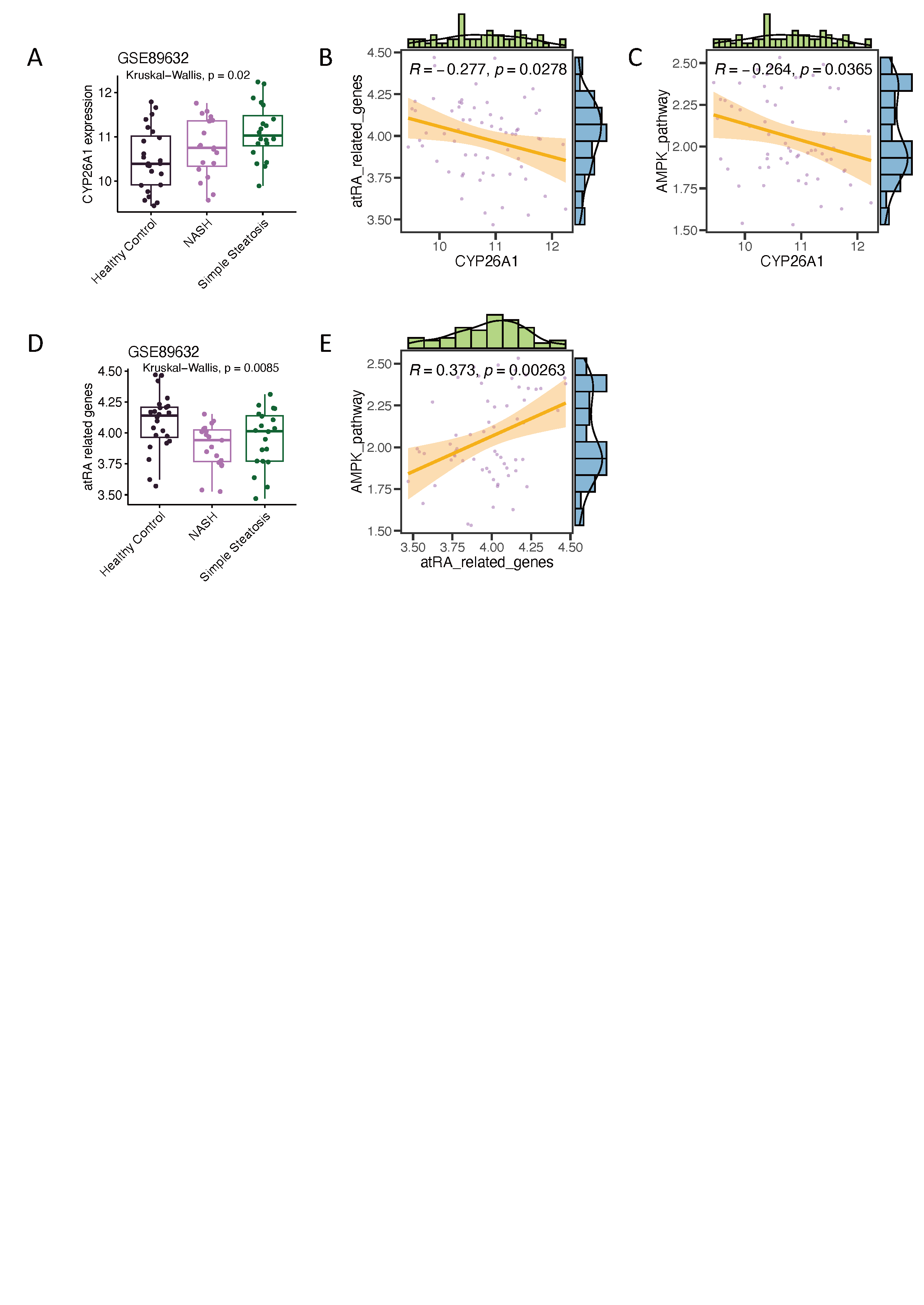


**Figure S8.** **Regulatory relation among CYP26A1, AMPK pathway and** **atRA-related genes in clinical patients with CLI.**

(A) Expression levels of CYP26A1 in GSE89632, which contains patients with NASH or simple steatosis. Detailed information could be found in "Methods and Materials" part. (B) Correlation analysis between CYP26A1 and atRA-related genes. Gene set score was estimated using ssGSEA algorithm. (C) Correlation analysis between CYP26A1 and AMPK pathway. The pathway score was calculated using ssGSEA algorithm. (D) Activation scores of atRA-related genes in GSE89632. (E) Correlation analysis between atRA-related genes and AMPK pathway. The gene set score was inferred using ssGSEA algorithm.


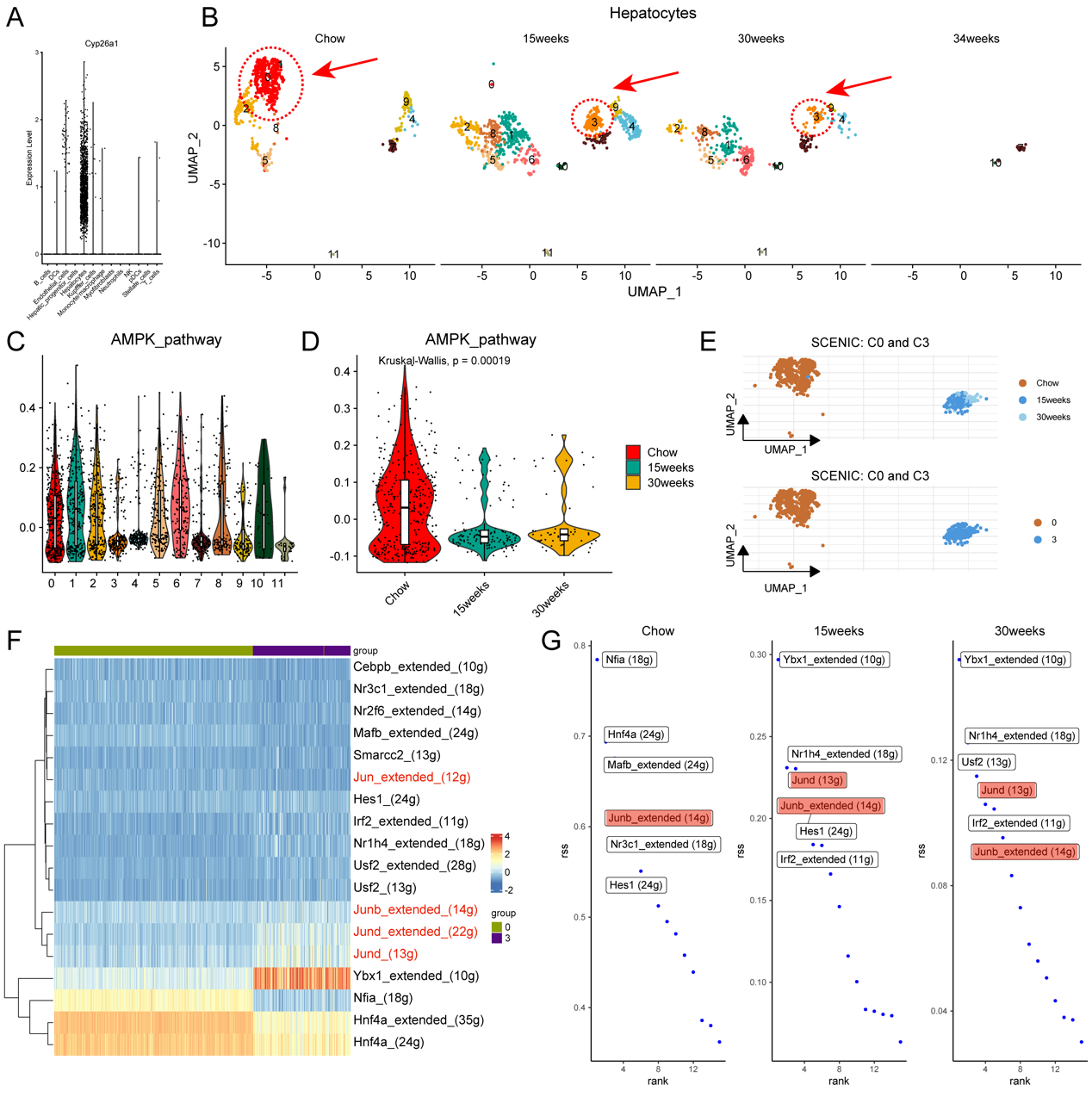


**Figure S9. SCENIC analysis of hepatocytes from NASH mouse.**

(A) Expression level of Cyp26a1 in various cell types of NASH mouse. (B) Subcluster analysis of hepatocytes in NASH mouse. (C) AMPK pathway score of hepatocyte clusters calculated by addmodule algorithm. (D) AMPK pathway score of different timepoint hepatocytes (C0 and C3) calculated by addmodule algorithm. (E) UMAP analysis of hepatocytes (C0 and C3). (F) SCENIC analysis of hepatocytes (C0 and C3) in NASH mouse. (G) Top TFs of hepatocytes (C0 and C3) in NASH mouse calculated by SCENIC analysis.

**A B**

**Figure S10. Cyp26a1 expression in mouse liver and primary mouse hepatocytes treated with atRA.**

**(A)** Analysis of Cyp26a1 expression after RT-PCR amplification of RNA from mouse liver samples shown in Fig. 5A. Gapdh was used as a housekeeping gene. Statistical significance was determined by unpaired two-tail t-test. *Error bars* represent S.D. ***P < 0.001.

**(B)** Analysis of Cyp26a1 expression after RT-PCR amplification of RNA from *Fah*^-/-^ mouse primary hepatocyte treated with atRA at indicated concentrations and 6 hours. Gapdh was used as a housekeeping gene. Statistical significance was determined by Ordinary one-way ANOVA. *Error bars* represent S.D.*P < 0.05; ***P < 0.001; NS: not significant.

**
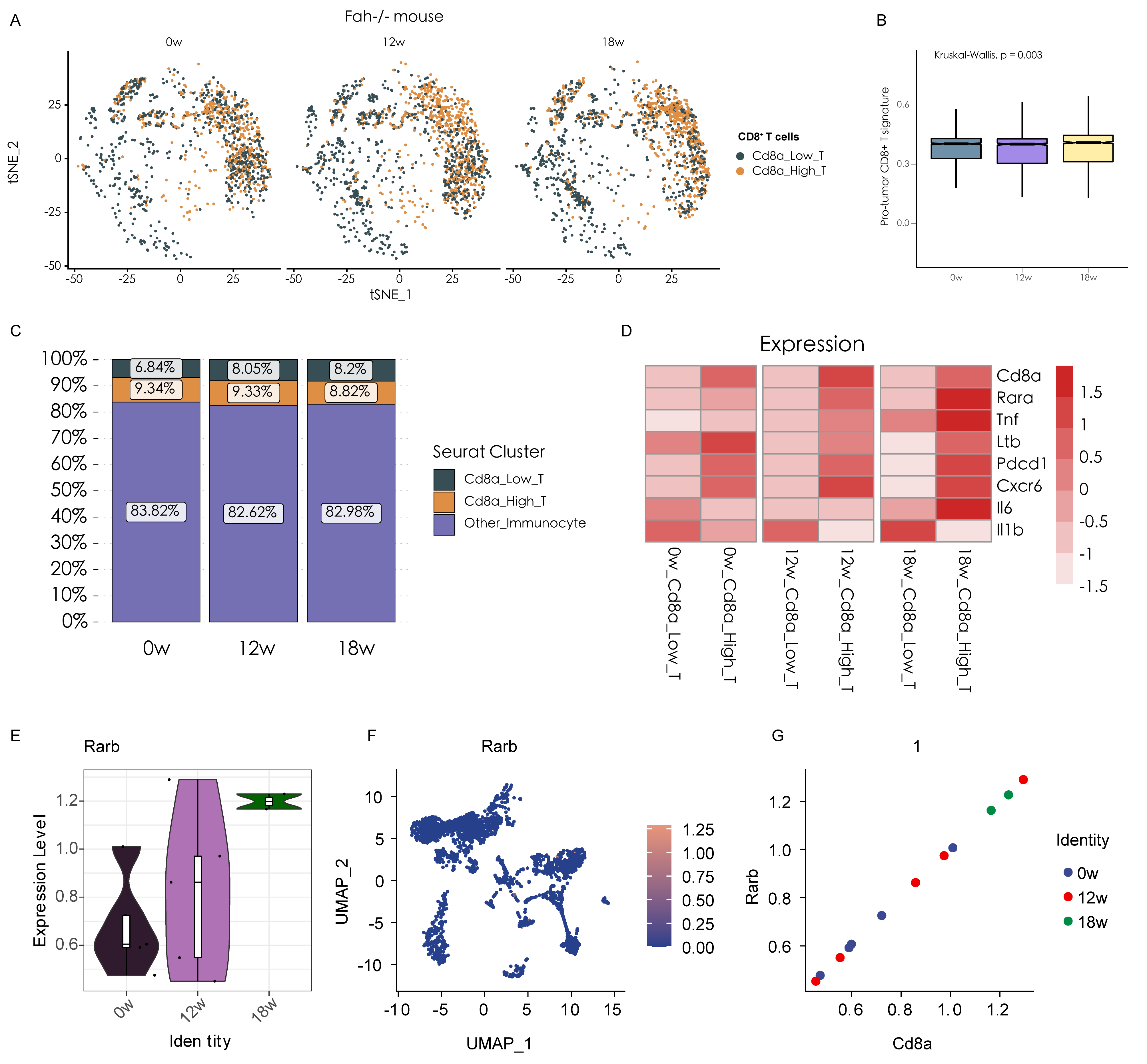
**

**Figure S11. The expression levels of CD8^+^ T cells in *Fah^-/-^* mouse at 0, 12, and 18 weeks**

(A) t-SNE visualization of Cd8^High^ T cell (grey) and Cd8^Low^ T cell group (orange) in *Fah^-/-^* mouse at 0, 12, and 18 weeks. (B) Expression levels of tumor promoting CD8^+^ T cell signature in *Fah^-/-^* mouse at 0, 12, and 18 weeks. (C) Histogram indicating the proportion of cells in the Cd8^High^ T cell (grey), Cd8^Low^ T cell (orange) and other immune types group (purple) at 0, 12, and 18 weeks. (D) Heatmap presenting the expression levels of Cd8a, Rara, Tnf, Ltb, Pdcd1, Cxcr6, Il6 and Il1b in the Cd8^High^ T cell and Cd8^Low^ T cell group at 0, 12, and 18 weeks. (E) Violin plot presenting the expression levels of Rarb at 0, 12, and 18 weeks. (F) UMAP plot showing the expression levels of Rarb. (G) Correlation analysis of Cd8a and Rarb in *Fah^-/-^* mouse at 0 (blue), 12 (red), and 18 weeks (green).

**
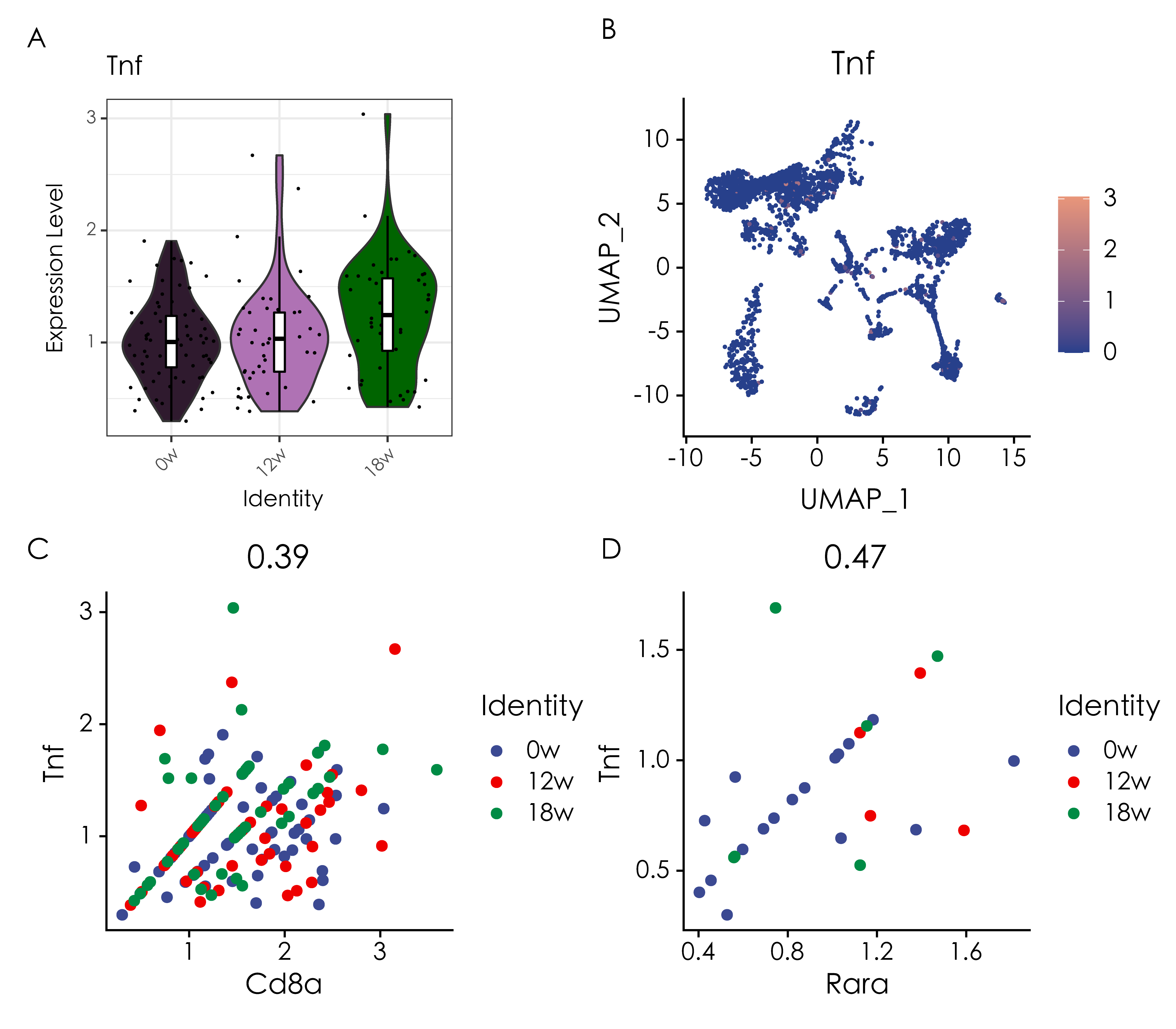
**

**Figure S12. The expression levels of Tnf and its correlation with Cd8a and Rara**

(A) Violin plot presenting the expression levels of Tnf at 0, 12, and 18 weeks. (B) UMAP plot showing the expression levels of Tnf. (C) Correlation analysis of Cd8a and Tnf in *Fah^-/-^* mouse at 0 (blue), 12 (red), and 18 weeks (green). (D) Correlation analysis of Rara and Tnf in *Fah^-/-^* mouse at 0 (blue), 12 (red), and 18 weeks (green).
