# Supplementary material for: Metformin reduces hepatocarcinogenesis by inducing downregulation of Cyp26a1 and CD8+ T cells
Source: Clin Transl Med. 2023 Nov 23;13(11):e1465. doi: 10.1002/ctm2.1465 (PMC10668005; doi:10.1002/ctm2.1465)
Supplement: Supplementary file 3 — Supporting Information [file CTM2-13-e1465-s002.docx]

**
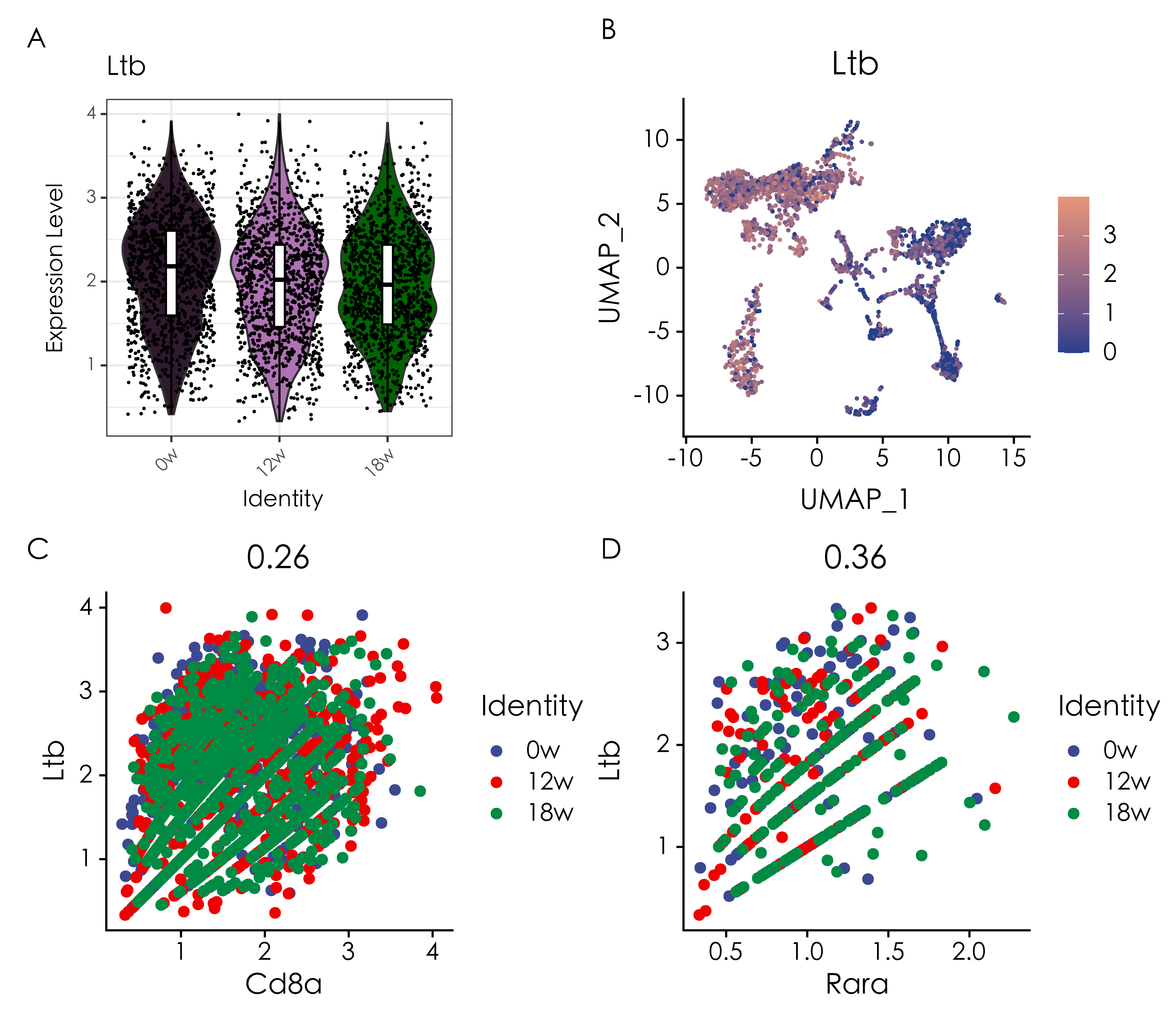
**

**Figure S13. The expression levels of Ltb and its correlation with Cd8a and Rara**

(A) Violin plot presenting the expression levels of Ltb at 0, 12, and 18 weeks. (B) UMAP plot showing the expression levels of Ltb. (C) Correlation analysis of Cd8a and Ltb in *Fah^-/-^* mouse at 0 (blue), 12 (red), and 18 weeks (green). (D) Correlation analysis of Rara and Ltb in *Fah^-/-^* mouse at 0 (blue), 12 (red), and 18 weeks (green).

**
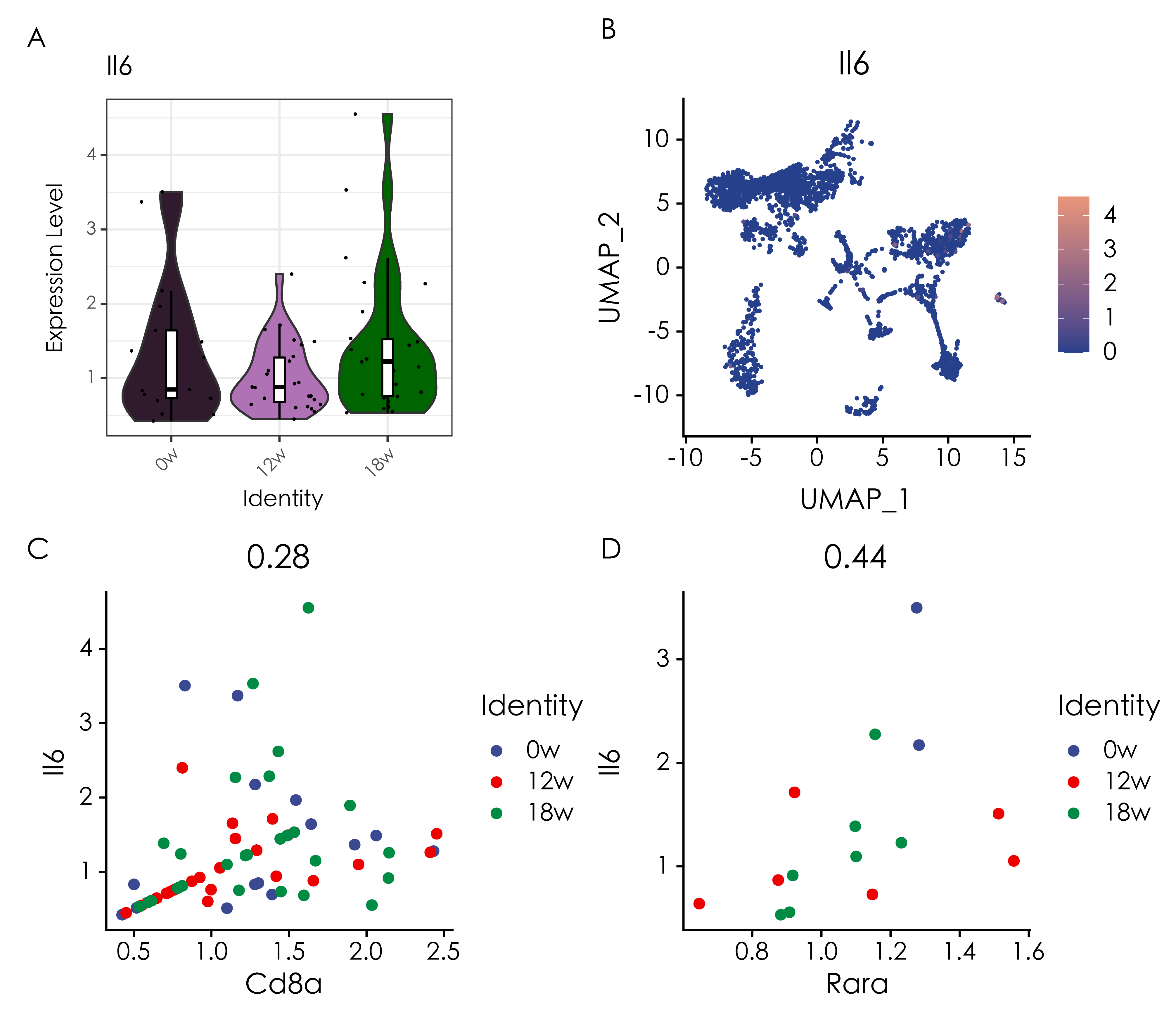
**

**Figure S14. The expression levels of Il6 and its correlation with Cd8a and Rara**

(A) Violin plot presenting the expression levels of Il6 at 0, 12, and 18 weeks. (B) UMAP plot showing the expression levels of Il6. (C) Correlation analysis of Cd8a and Il6 in *Fah^-/-^* mouse at 0 (blue), 12 (red), and 18 weeks (green). (D) Correlation analysis of Rara and Il6 in *Fah^-/-^* mouse at 0 (blue), 12 (red), and 18 weeks (green).

**
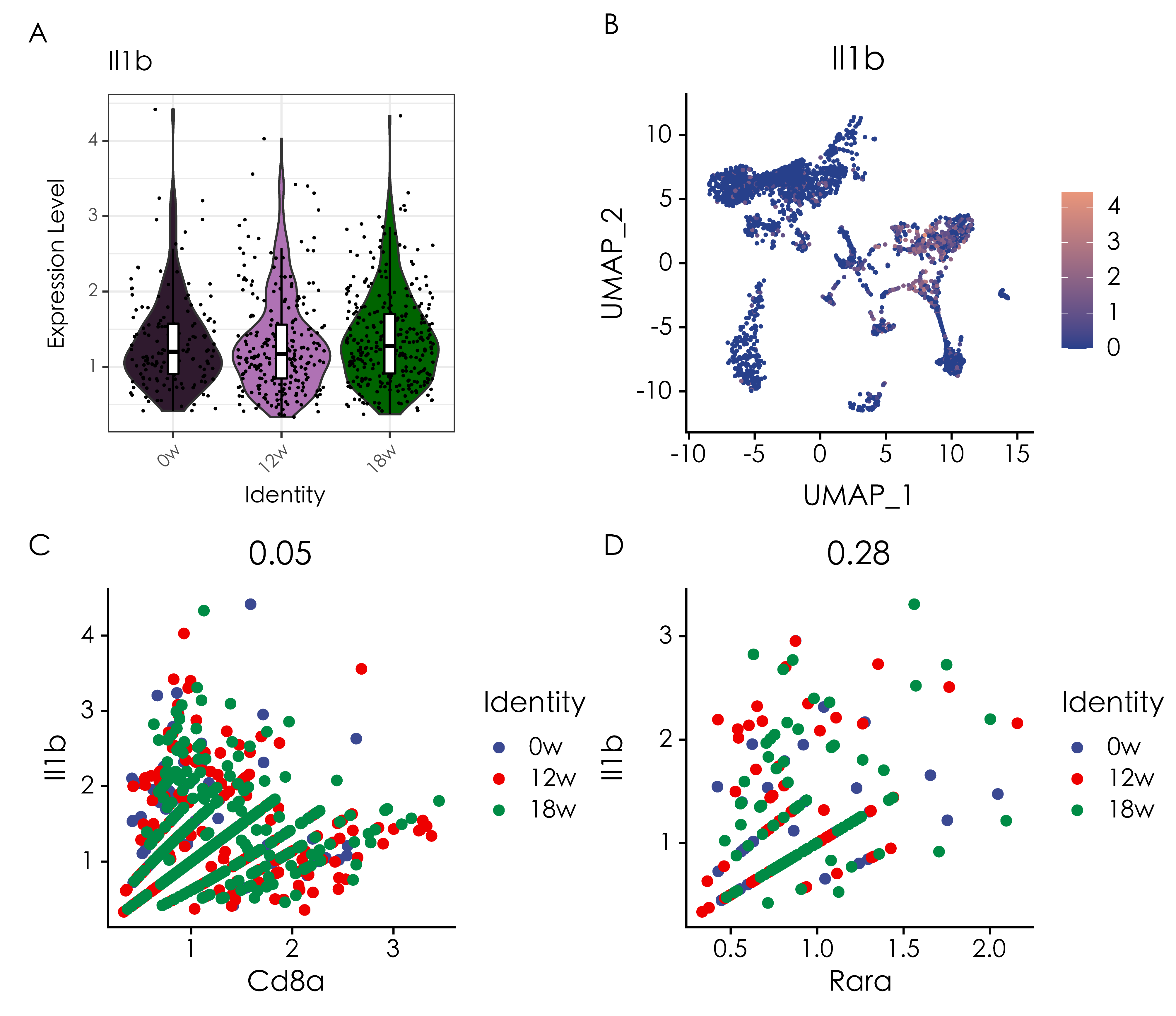
**

**Figure S15. The expression levels of Il1b and its correlation with Cd8a and Rara**

(A) Violin plot presenting the expression levels of Il1b at 0, 12, and 18 weeks. (B) UMAP plot showing the expression levels of Il1b. (C) Correlation analysis of Cd8a and Il1b in *Fah^-/-^* mouse at 0 (blue), 12 (red), and 18 weeks (green). (D) Correlation analysis of Rara and Il1b in *Fah^-/-^* mouse at 0 (blue), 12 (red), and 18 weeks (green).

**
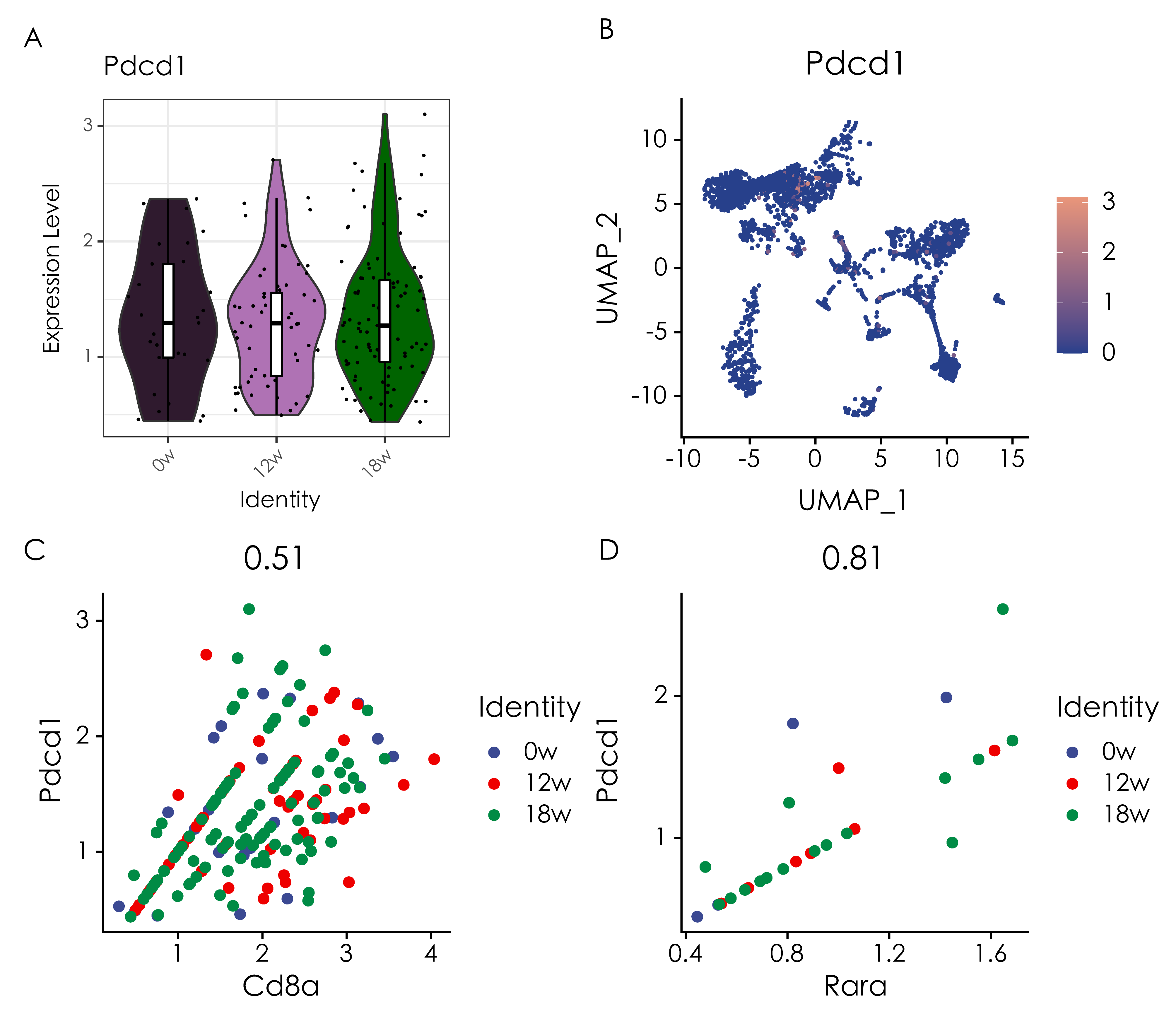
**

**Figure S16. The expression levels of Pdcd1 and its correlation with Cd8a and Rara**

(A) Violin plot presenting the expression levels of Pdcd1 at 0, 12, and 18 weeks. (B) UMAP plot showing the expression levels of Pdcd1. (C) Correlation analysis of Cd8a and Pdcd1 in *Fah^-/-^* mouse at 0 (blue), 12 (red), and 18 weeks (green). (D) Correlation analysis of Rara and Pdcd1 in *Fah^-/-^* mouse at 0 (blue), 12 (red), and 18 weeks (green).

**
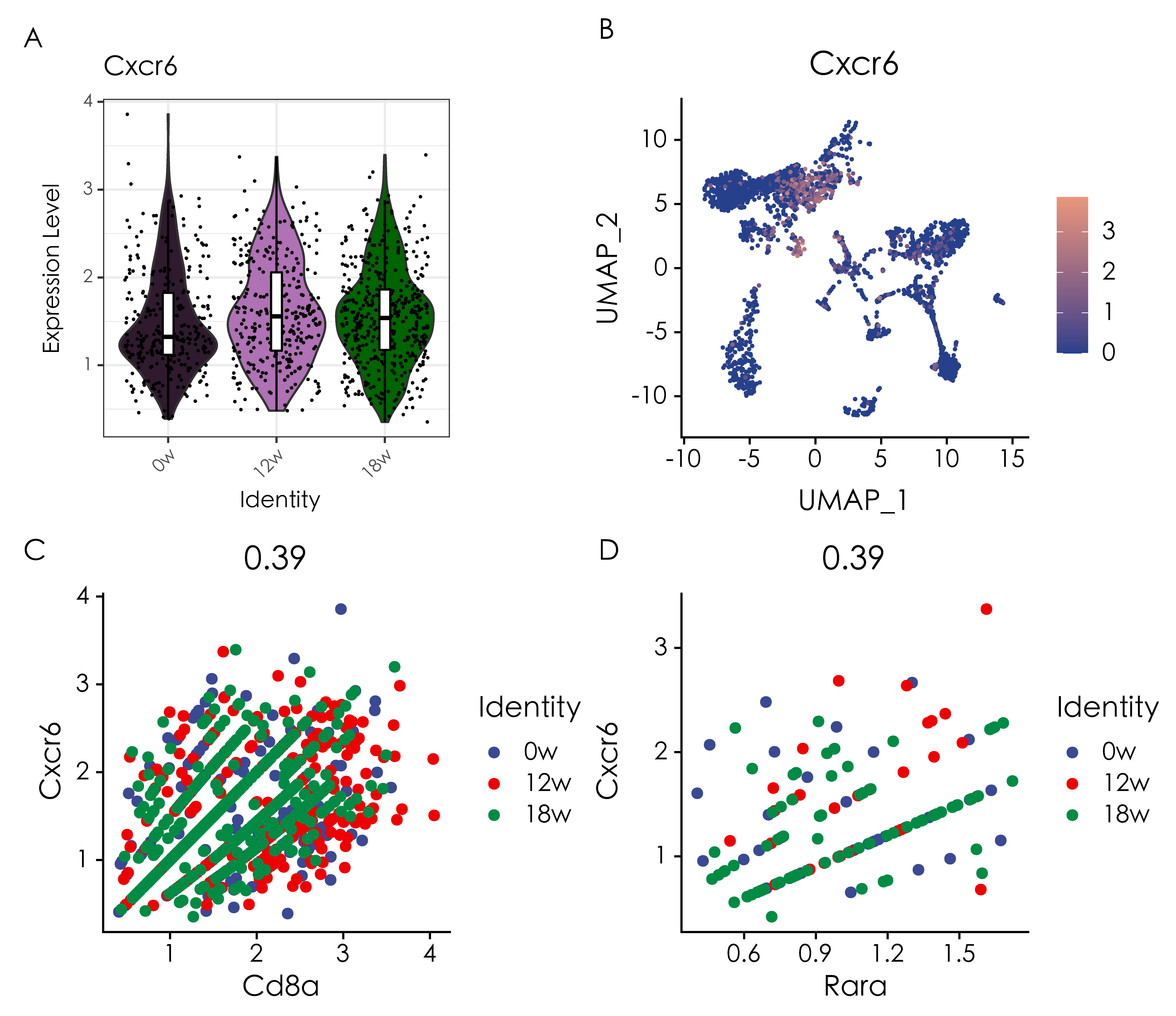
**

**Figure S17. The expression levels of Cxcr6 and its correlation with Cd8a and Rara**

1. Violin plot presenting the expression levels of Cxcr6 at 0, 12, and 18 weeks. (B) UMAP

plot showing the expression levels of Cxcr6. (C) Correlation analysis of Cd8a and Cxcr6 in *Fah^-/-^* mouse at 0 (blue), 12 (red), and 18 weeks (green). (D) Correlation analysis of Rara and Cxcr6 in *Fah^-/-^* mouse at 0 (blue), 12 (red), and 18 weeks (green).
