# Supplementary material for: Metformin reduces hepatocarcinogenesis by inducing downregulation of Cyp26a1 and CD8+ T cells
Source: Clin Transl Med. 2023 Nov 23;13(11):e1465. doi: 10.1002/ctm2.1465 (PMC10668005; doi:10.1002/ctm2.1465)
Supplement: Supplementary file 4 — Supporting Information [file CTM2-13-e1465-s004.docx]

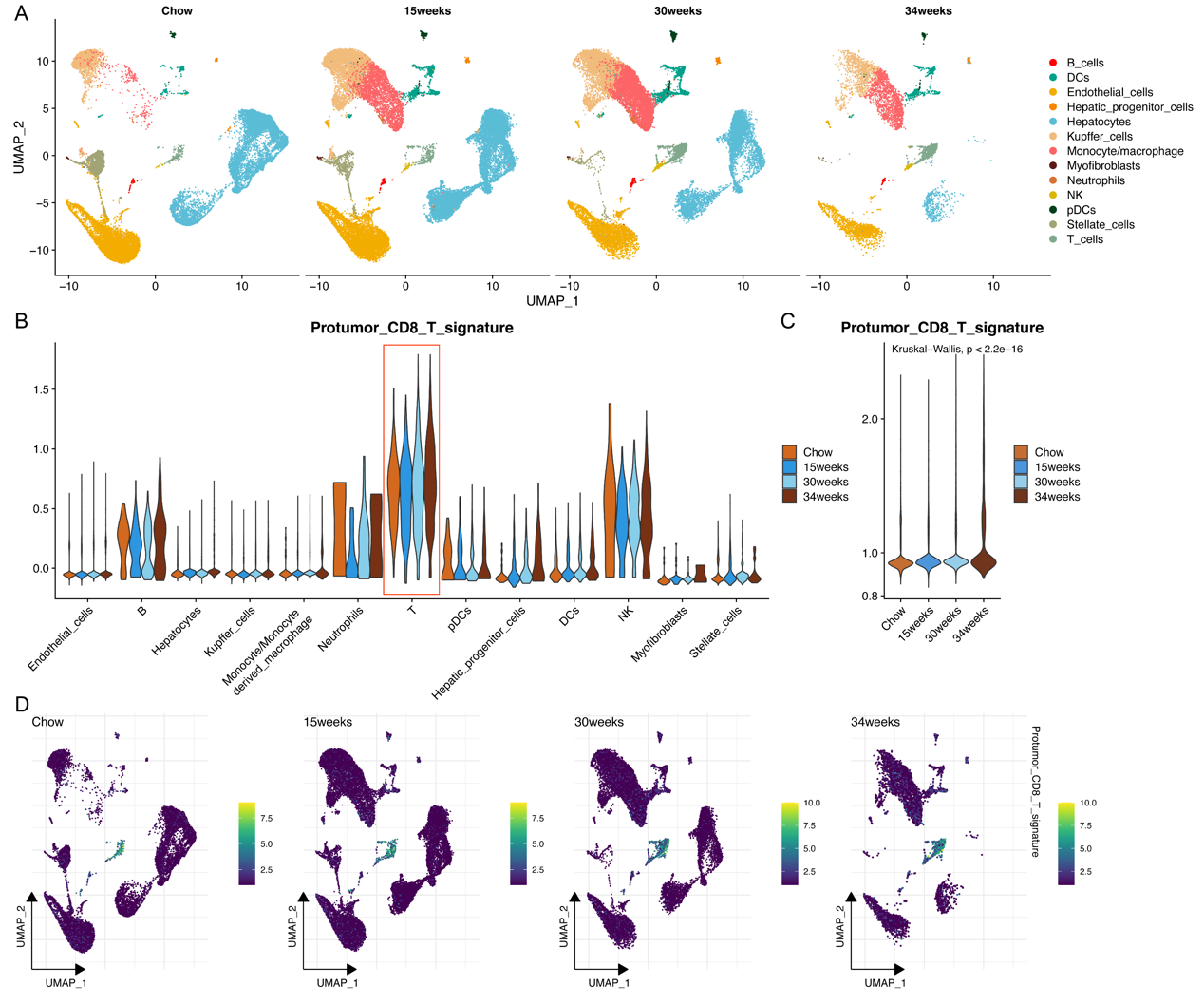


**Figure S18. Analysis of the protumor CD8 T signature in NASH mouse.**

(A) Distribution of various cell types of NASH mouse. (B) Activation score of protumor CD8 T signature in different cell types. (C) Activation score of protumor CD8 T signature in different timepoints. (D) Distribution of protumor CD8 T signature score in NASH mouse.

**
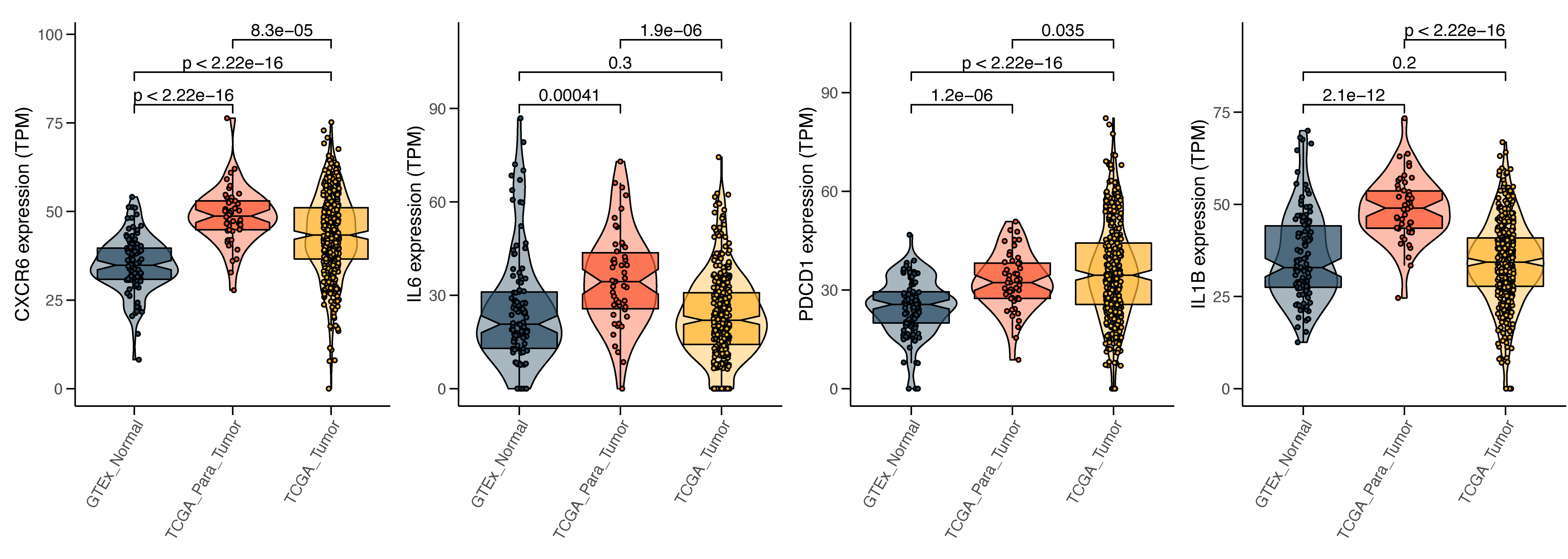
**

**Figure S19. The expression characteristics of CD8+ T cells in** **GTEx normal, TCGA para-tumor and TCGA tumors tissues**

Violin plot presenting the expression levels of CXCR6, IL6, PDCD1 and IL1B in GTEx normal, TCGA para-tumor and TCGA tumors tissues.

**
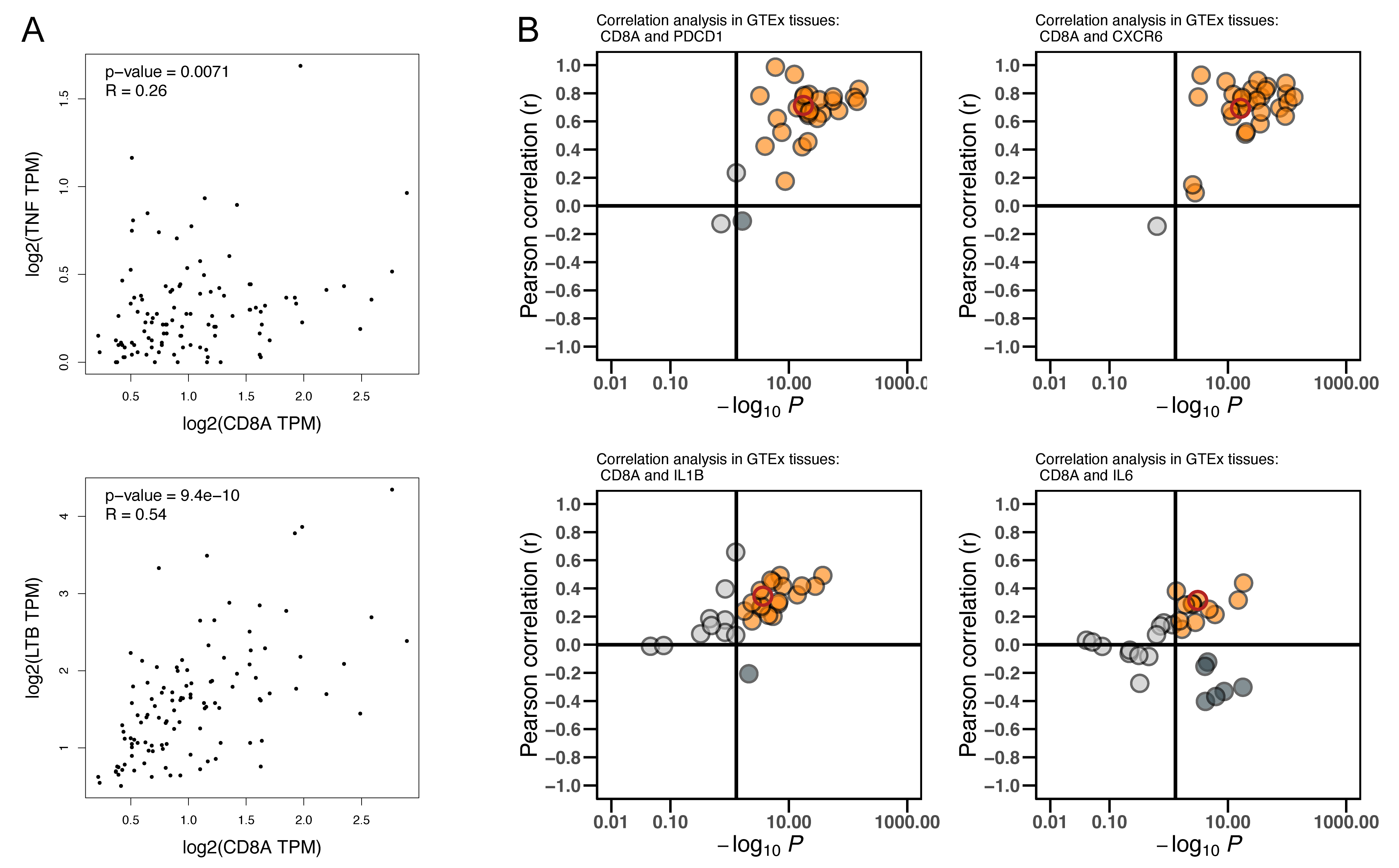
**

**Figure S20. CD8A is positively correlated with pro-tumor cytokines in GTEx normal tissues**

1. Correlation analysis of CD8A and TNF and LTB in 30 GTEx normal tissues.

(B) Correlation analysis of CD8A and PDCD1, CXCR6, IL6, IL1B in 30 GTEx normal tissues.

**
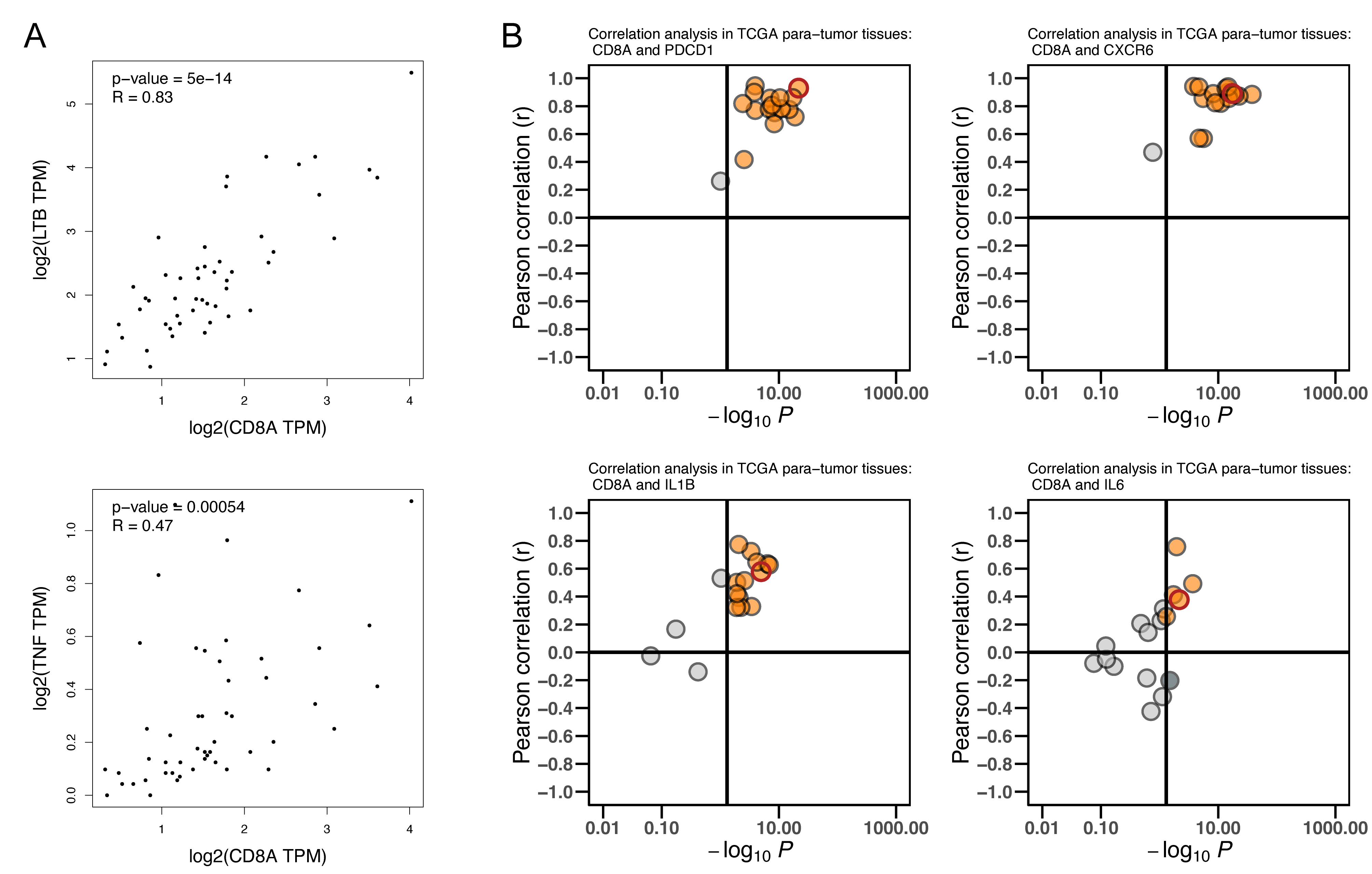
**

**Figure S21. CD8A is positively correlated with pro-tumor cytokines in TCGA para-tumor tissues**

1. Correlation analysis of CD8A and TNF and LTB in 17 TCGA para-tumor tissues.

(B) Correlation analysis of CD8A and PDCD1, CXCR6, IL6, IL1B in 17 TCGA para-tumor tissues.

**
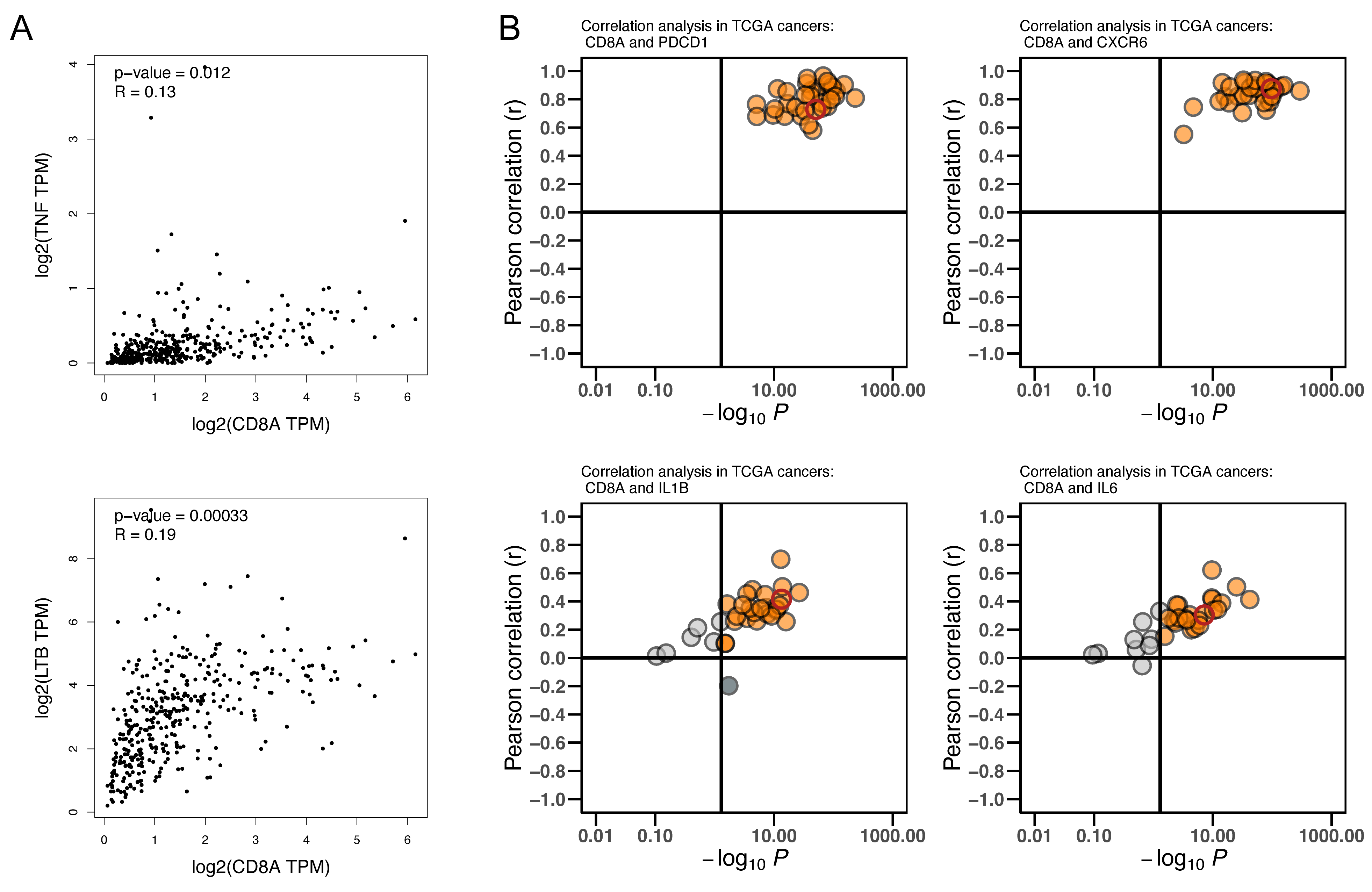
**

**Figure S22. CD8A is positively correlated with pro-tumor cytokines in TCGA tumors tissues**

(A) Correlation analysis of CD8A and TNF and LTB in 34 TCGA tumors tissues.

(B) Correlation analysis of CD8A and PDCD1, CXCR6, IL6, IL1B in 34 TCGA tumors tissues.
